# Using spectral characterization to identify healthcare-associated infection (HAI) patients for clinical contact precaution

**DOI:** 10.1038/s41598-023-41852-5

**Published:** 2023-09-27

**Authors:** Jiaming Cui, Sungjun Cho, Methun Kamruzzaman, Matthew Bielskas, Anil Vullikanti, B. Aditya Prakash

**Affiliations:** 1 College of Computing, Georgia Institute of Technology, Atlanta, GA 30332 USA; 2 Biocomplexity Institute, University of Virginia, Charlottesville, VA 22904 USA; 3 Department of Computer Science, University of Virginia, Charlottesville, VA 22904 USA

**Keywords:** Computational models, Infectious diseases

## Abstract

Healthcare-associated infections (HAIs) are a major problem in hospital infection control. Although HAIs can be suppressed using contact precautions, such precautions are expensive, and we can only apply them to a small fraction of patients (i.e., a limited budget). In this work, we focus on two clinical problems arising from the limited budget: (a) choosing the best patients to be placed under precaution given a limited budget to minimize the spread (the isolation problem), and (b) choosing the best patients to release when limited budget requires some of the patients to be cleared from precaution (the clearance problem). A critical challenge in addressing them is that HAIs have multiple transmission pathways such that locations can also accumulate ‘load’ and spread the disease. One of the most common practices when placing patients under contact precautions is the regular clearance of pathogen loads. However, standard propagation models like independent cascade (IC)/susceptible-infectious-susceptible (SIS) cannot capture such mechanisms directly. Hence to account for this challenge, using non-linear system theory, we develop a novel spectral characterization of a recently proposed pathogen load based model, 2-Mode-SIS model, on people/location networks to capture spread dynamics of HAIs. We formulate the two clinical problems using this spectral characterization and develop effective and efficient algorithms for them. Our experiments show that our methods outperform several natural structural and clinical approaches on real-world hospital testbeds and pick meaningful solutions.

## Introduction

Healthcare-associated infections (HAIs) such as Methicillin-resistant *Staphylococcus aureus* (MRSA) and *Clostridioides difficile* (*C. difficile*) are infections acquired by patients during treatment at healthcare facilities such as hospitals and long-term care homes^1–3^. While under treatment, patients can get exposed to pathogens from other sick patients or contaminated locations like rooms or medical devices. HAIs impose tremendous costs on hospitals, as they tend to extend patients’ stays longer than initially planned to reach full recovery and increase the risk of mortality^4–9^. Due to its high health burden, epidemiologists and clinicians have proposed several approaches to control HAI spread in hospitals, such as contact precautions, antimicrobial stewardship, and contact tracing^10–15^. Among these approaches, contact precautions have been shown to be more effective in preventing further nosocomial infections^16, 17^, and easier to implement^18^. Therefore, they are most commonly used in most hospitals to control HAI spread^12^. However, real-world contact precautions are expensive^7, 19–23^ and therefore they can only be applied to a small fraction of patients (a ‘budget’). Further, there are several downsides to contact precautions on patient health outcomes, e.g.,^22, 23^. Therefore, using contact precautions optimally is an important goal. We consider the following two important problems in HAI control with a limited budget, motivated by the above considerations.

**Figure 1:**
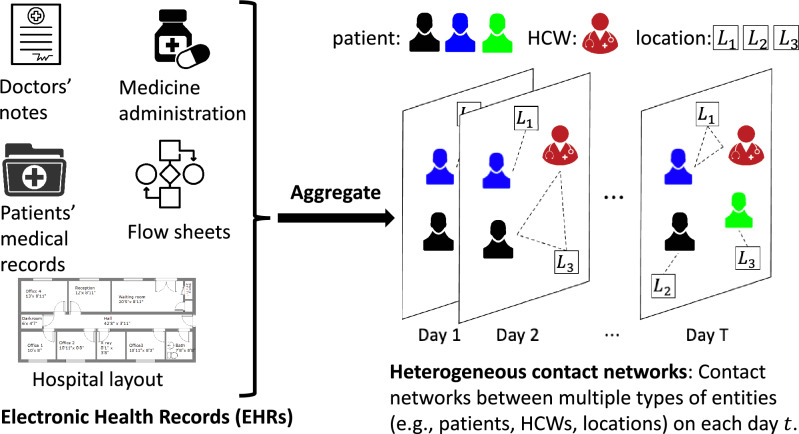
In this work, we collect the time and location of interactions between patients and HCWs from different tables in the electronic health records (EHRs) dataset, such as medicine administration, admission and discharge and flow sheets, and doctors’ notes. We construct daily heterogeneous contact networks from this data (see more details in the Supplementary Information). For example, as shown in the figure on day *T*, the red healthcare worker and blue patient are in location $L_1$, the black patient is in location $L_2$, and the green patient is in location $L_3$.

### Clinical Problem 1

(Isolation Problem, IP) Given a limited budget for precaution, how can we choose the “best” patients to put under precaution?

### Clinical Problem 2

(Clearance Problem, CP) Assume a hospital initially has a set of patients under precaution, but limited budget requires a subset of these patients to be cleared from precaution. How can we choose the “best” patients to release from precaution?

Intuitively, IP aims to find which patients to put under precautions. In contrast to IP, the setting for CP assumes that a set of patients are already under precautions, and the goal is to find a subset of patients from this set to *clear* from precautions. Although there are some guidelines on removing patients from contact precautions^18, 24^ from clinical perspective, this is still a novel problem in the network science research field in terms of problem formulation and optimization: it is the opposite of the well-studied influence maximization problem on social networks^25^ since the objective of CP is to minimize the spread while taking off precautions from nodes. This problem is also very different from the influence containment problem^26, 27^ since these problems are edge-based, while IP and CP need to remove multiple edges when only isolating/releasing one node. No good formulations exist for the clinical problems above, as we cannot easily assess how each precaution on a specific patient affects the overall epidemic.

It is important to note that healthcare workers (HCWs) and contaminated physical surfaces can also spread the pathogens^28–30^ in addition to the direct patient-patient contacts. However, traditional contact networks with only patient-patient contacts cannot capture such pairwise relations among diverse entities. To account for this challenge, heterogeneous contact networks are needed in modeling. Heterogeneous graphs contain multiple types of entities and can represent the diversity among nodes/pairwise relations in real-life networks. They have gathered great interest in recent years with applications in personalized recommendation^31^, publication ranking^32^, and drug design^33^. Here, we consider heterogeneous contact networks where each node can be one of three types: patients, HCWs, and locations. Besides, the contacts between each node are also changing with time. Hence, such heterogeneous contact networks should also be time-varying to capture the contact changes with time. We note that contact networks are not explicitly known, and need to be inferred. As shown in Fig. 1, we infer a contact network from Electronic Health Records (EHRs) data, which includes a variety of patients’ medical records and doctors’ notes. Specifically, we infer interactions between HCWs and patients through medicine administration, admission and discharge and flow sheets, and doctor notes from EHRs, and aggregate them into daily heterogeneous contact networks for each day *t*. See Supplementary Information for more details.

**Figure 2:**
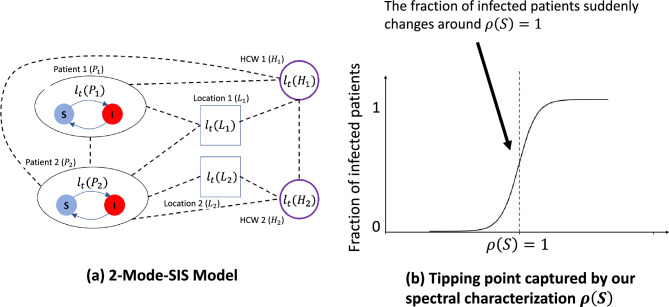
(**a**) An example of the 2-Mode-SIS model on a heterogeneous contact network: on some day *t*, there are two patients ($P_1$, $P_2$), two HCWs ($H_1$, $H_2$), and two locations ($L_1$, $L_2$) in the network, and dash lines represent edges in the heterogeneous contact network (e.g., $P_1$ was in $L_1$ on day *t* as well as in contact with $P_2$ and $H_1$ on day *t*). For every node *i*, their pathogen load is represented as ${\varvec{l}}_t(i)$ by 2-Mode-SIS model, and these loads can spread via contacts. For patients $P_1$, $P_2$, their infection states can transfer between *S* (${\varvec{x}}_t(i) = 0$) and *I* (${\varvec{x}}_t(i) = 1$). See more details in the paper and Supplementary Information. (**b**) Our spectral characterization $\rho ({\varvec{S}})$ captures a critical “tipping point”: When $\rho ({\varvec{S}})>1$, the fraction of infected patients will be close to 1. When $\rho ({\varvec{S}})<1$, almost none of the patients will be infected (i.e., the epidemic dies out).

Modeling on heterogeneous contact networks is different from modeling on traditional contact networks. We need to identify the role of HCWs and location nodes in spreading the pathogens and involve this in modeling. We assume that HCWs and locations can also carry some pathogen loads and spread them to patients, but HCWs do not get infected. In this paper, we use a recently proposed model for HAI, the 2-Mode-SIS model proposed by Jang et al.^34^, which incorporates pathogens spread through both people and locations as shown in Fig. 2a. Such pathogen load based models have been used in recent high-profile HAI modeling studies^13, 35–37^. Importantly, they can also model the cleaning practice widely used in contact precautions (e.g., using disinfectants to clean the ward) directly^38–42^ compared with traditional independent cascade (IC)^43^/susceptible-infectious-susceptible (SIS)^44^ models. However, modeling with 2-Mode-SIS model is non-trivial since there are multiple transmission pathways through multiple kinds of nodes, making its dynamics harder to model.

The main idea of this paper is to use spectral characterization to help formulate the two clinical problems we are interested in as network optimization problems: We give a novel spectral characterization $\rho ({\varvec{S}})$ of the 2-Mode-SIS model on underlying time-varying people-location heterogeneous contact networks using stability analysis of discrete-time non-linear dynamical systems^45^. We first approximate the propagation dynamics of the 2-Mode-SIS model on heterogeneous networks, after which we can analyze the stability of its equilibrium point (specifically, *all-zero* point where the disease dies out) and check whether this point is stable or not. As shown in Fig. 2b, we show that this characterization is meaningful in capturing a ‘tipping point’ for HAI spread dynamics. At this “tipping point”, there will be a phase transition for the fraction of infected patients at the end: when $\rho ({\varvec{S}})>1$, the fraction of infected patients will be close to 1. When $\rho ({\varvec{S}})<1$, almost none of the patients will be infected (i.e., the epidemic dies out). This $\rho ({\varvec{S}})$ is similar to the basic reproductive number $R_0$^46–49^ for standard SIR-type models, and helps relate the structural and disease parameters to the dynamics. In the following section, we will show how we formulate the clinical problems in detail, and propose our Greedy-Spectral algorithm to address it.

## Methods

### 2-Mode-SIS model

As shown in Fig. 2a, we use the 2-Mode-SIS model^34^ to simulate the spread of pathogens (see Procedure 1 for simulation steps). 2-Mode-SIS is pathogen ‘load-based’ where the patient can be either *susceptible* or *infected*, and the model keeps track of pathogen load on all people and locations using a load vector ${\varvec{l}}_t$. Unlike classical SIS models^50^, in 2-Mode-SIS model, the probability of a patient *i* to get infected from susceptible is formulated as a dose-response function proportional to the amount of pathogen on the patient *i*, or ${\varvec{l}}_t(i)$, as shown in Procedure 1 step 8 with $\beta$ as the disease infectivity). Once infected, the patient *i* sheds $\alpha$ additional pathogen per timestep to his own load, which can later be transferred to neighbors (both people and locations) via edges; this shedding continues until the patient recovers with recovery probability $\delta$. Specifically, in this model, each node carries some amount of pathogens that changes over time, and the exchange of pathogens among the nodes is driven by edges that imply close contact among the nodes. 2-Mode-SIS model uses $\tau _{ijt}$ to denote the ratio of pathogen being transferred (or remaining if $i = j$) from node *j* to node *i* at time *t*, and $\phi _{i}$ to denote the natural pathogen reduction rate on node *i*. By constructing a transfer matrix ${\varvec{R}}_t$ from the transfer ratios $\tau _{ijt}$, reduction rates $\phi _{i}$, and adjacency matrix ${\varvec{A}}_t$, the pathogen load updates can be written as a linear operation as in Procedure 1 step 5. Here, ${\varvec{x}}_t$ is the infection state vector for all patients at time *t*, where the *i*th element, ${\varvec{x}}_t(i)$, corresponds to patient *i*. $\alpha$ is pathogen shedding rate for infected patients. Note that the column-sums of ${\varvec{R}}_t$ are restricted to be less than or equal to 1, which implies that the total amount of pathogen cannot increase after transfer (i.e., $\Vert {\varvec{R}}_t {\varvec{l}}_t\Vert _1 \le \Vert {\varvec{l}}_t\Vert _1$). Susceptible patients may still be colonized with pathogen loads and can spread them to others. HCWs and locations are assumed to be non-infectable, but act as pathways of pathogen transfer. More details are provided in Supplementary Information.

### Spectral characterization for the 2-Mode-SIS model

#### Approximation via NLDS

We develop a characterization to capture the overall dynamics of the system and derive a spectral characterization for the 2-Mode-SIS model. Our proposed approach is to approximate the propagation dynamics of the 2-Mode-SIS model using the stability analysis of discrete-time non-linear dynamical systems (NLDS) with two coupled states: the pathogen loads ${\varvec{l}}_t$ and the expectation of infection probabilities ${\varvec{p}}_t={\mathbb {E}}[{\varvec{x}}_t]$. Let $${\varvec{s}}_{t} = \begin{bmatrix} {\varvec{p}}_{t}\\ {\varvec{l}}_{t}\\ \end{bmatrix} \in {\mathbb {R}}^{(P+N)}$$ be the vector describing the state of the system at timestep *t*, we can write down the state transition updates at *t* and use linearity of expectation to get the vector for next timestep as ${\varvec{s}}_{t+1} = g_t \left( {\varvec{s}}_{t}\right)$, where $g_t$ is a non-linear mapping.

$$\begin{aligned} {\varvec{s}}_{t+1} = g_t \left( {\varvec{s}}_{t} \right) {:}{=}\begin{bmatrix} (1 - \delta ) {\varvec{p}}_{t} + \beta {\varvec{l}}_{t}(1 - {\varvec{p}}_{t})\\ {\varvec{R}}_1 {\varvec{l}}_{t} + \alpha {\varvec{p}}_{t}\\ \end{bmatrix} \end{aligned}$$

#### Stability analysis of the NLDS

With the non-linear equations of our approximated discrete-time NLDS, we can then analyze its asymptotic behavior. The long-term behavior of a dynamical system is dictated by the stability of its equilibrium points, at which the states no longer change over time (i.e., ${\varvec{s}}^{eq} = g({\varvec{s}}^{eq})$). The particular equilibrium point of interest is the *all-zero* point ${\varvec{0}}$ where there is no infection as well as no pathogen remaining. Clearly, all $g_t$ map ${\varvec{0}}$ onto itself and ${\varvec{0}}$ is an equilibrium point. Specifically, we write the Jacobian matrix evaluated at ${\varvec{0}}$ as ${\varvec{S}}_t$

$$\user2{S}_{t} = \left. {\nabla _{g} } \right|_{{\user2{S}_{t} = 0}} = \left. {\frac{{\partial _{{\user2{S}_{{t + 1}} }} }}{{\partial _{{\user2{S}_{t} }} }}} \right|_{{\user2{S}_{t} = 0}} = \left[ {\begin{array}{*{20}c} {\left( {1 - \delta } \right)\user2{I}_{P} } & {\beta \user2{I}_{P} } & 0 \\ {\alpha \user2{I}_{P} } & {\user2{R}_{1} } & {} \\ {\mathbf{0}} & {} & {} \\ \end{array} } \right]$$

Here, $I_P$ represents the identity matrix sized $P \times P$. The following Theorem 1 on asymptotic stability in a discrete-time NLDS shows what condition this equilibrium point is ‘stable’^45^, i.e. a small perturbation does not cause large deviations (see Supplementary Information for the proof).
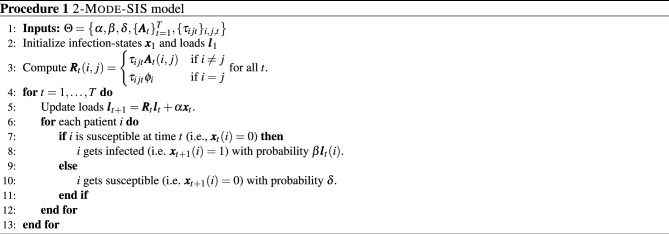


##### Theorem 1

(Spectral characterization) If the spectral radius (i.e., the largest eigenvalue) of the system matrix ${\varvec{S}} = \prod _{t=1}^T {\varvec{S}}_{T - t + 1}$ is less than 1 (i.e. $\rho ({\varvec{S}}) < 1$), then the all-zero equillibrium of the NLDS is asymptotically stable.

Theorem 1 demonstrates that $\rho ({\varvec{S}}) = 1$ is a critical tipping point that there will be a phase transition for the fraction of infected patients at the end: when $\rho ({\varvec{S}}) > 1$, there is always a fraction of infected patients. When $\rho ({\varvec{S}}) < 1$, almost no patients will be infected (i.e., the epidemic dies out). The tipping point behavior is similar to that associated with the basic reproductive number $R_0$. The characterization in Theorem 1 also helps explain the counter-intuitive ‘dilution effect’ (i.e., the number of infections decreases with increasing link probability between patients) observed in many such load-based models^34, 51, 52^. More explanations and an intuitive example are provided in Supplementary Information.

### Applications of $\rho ($*S*) for clinical problems

#### Using $\rho ($*S*) to formalize clinical problems

Next, we show how our spectral characterization $\rho ({\varvec{S}})$ can help formulate the two clinical problems IP and CP. The intuition is that our spectral characterization well captures the transition dynamics of the HAI breakout, and a lower $\rho ({\varvec{S}})$ value should correspond to fewer infections. We first formalize the IP problem as follows:

##### Formal Isolation Problem (FIP)

Let ${\varvec{S}}(\Theta , P)$ denote the system matrix given input 2-Mode-SIS model parameters $\Theta$ (e.g., $\alpha$, $\beta$, $\delta$ $\cdots$) and patients in set *P* under precaution. Given all patients *X* and a budget constraint *k*, find the subset of patients $P^*$ for precaution s.t.

$$\begin{aligned} P^* = \mathop {{{\,\mathrm{arg\,min}\,}}}\limits _{P} \rho ({\varvec{S}}(\Theta , P)) \text { subject to } P \subset X \text { and } |P| = k \end{aligned}$$

This formulation uses $\rho ({\varvec{S}}(\Theta , P))$ to capture how isolating patients in *P* will affect the epidemic. Here, we formally model bringing a patient *i* under precaution by zeroing out off-diagonal terms in the *i*-th row and column in each pathogen transfer matrix ${\varvec{R}}_t$ (i.e., cut off its links with other nodes), and reducing the diagonal terms ${\varvec{R}}_{t}(i,i)$ by a multiplicative factor $\phi _{ppe}<1$ (i.e., reduce its remaining pathogen via cleaning). They are widely used together in hospitals for contact precautions^11, 18, 53^. Note that the current setup can be readily modified to capture other contact precaution practices while maintaining a similar problem formulation. Similarly, we can also formalize the CP problem:

##### Formal Clearance Problem (FCP)

Let ${\varvec{S}}(\mathcal {\cdot })$ be defined as in FIP. Given an initial set *Q* of *d* candidate patients already under precaution ($| Q | = d$) and a budget constraint $k < d$, find the subset of patients $P^*$ for clearance s.t.

$$\begin{aligned} P^* = \mathop {{{\,\mathrm{arg\,min}\,}}}\limits _{P} \rho ({\varvec{S}}(\Theta ,Q \setminus P)) \text { subject to } P \subset Q \text { and } |P| = k \end{aligned}$$

### Greedy-spectral algorithm

#### Numerical issue in computing $\rho ({\varvec{S}})$

A natural algorithm for FIP or FCP would try adding (or removing) each candidate patient *P* under (or from) precaution and then compute by how much $\rho ({\varvec{S}})$ changes to incrementally build a feasible solution set while minimizing $\rho ({\varvec{S}})$. In practice, however, we find that computing the change in $\rho ({\varvec{S}})$ due to a single node removal runs into numerical issues: if there are much more patient-to-non-patient interactions than patient-to-patient interactions, the change in $\rho ({\varvec{S}})$ w.r.t. *P* can be too small to be computed within finite precision. The issue even gets worse when the total number of nodes *N* and the number of timesteps *T* increase (see Supplementary Information for a detailed explanation).

#### Alternative strategy and final algorithm

Hence we propose an alternate strategy to solve FIP and FCP by minimizing an upper bound on $\rho ({\varvec{S}})$ that can be computed more precisely. In particular, we define a *system-adjacency* matrix as ${\varvec{A}}{:}{=}\prod _{t=1}^T {\varvec{A}}_{T - t + 1}$, where ${\varvec{A}}_{t}$ is the adjacency matrix for time *t*, and minimize its the largest eigenvalue $\rho ({\varvec{A}})$ as a surrogate to minimize the upper bound of $\rho ({\varvec{S}})$. The following theorem shows that $\rho ({\varvec{S}})$ is upper bounded by a *monotonic* function of $\rho ({\varvec{A}})$, justifying our approach of minimizing $\rho ({\varvec{A}})$ instead. We provide a detailed proof for Theorem 2 in Supplementary Information.

##### Theorem 2

Let ${\varvec{S}}$, ${\varvec{A}}$ denote the system and system-adjacency matrices of 2-Mode-SIS model with *T* number of timesteps. Then $\rho ({\varvec{S}})$ is upper bounded by a monotone function *f* of $\rho ({\varvec{A}})$.

Fortunately, $\rho ({\varvec{A}})$ does not suffer from the same numerical issue as $\rho ({\varvec{S}})$. Therefore, we propose a simple yet effective heuristic algorithm, Greedy-Spectral, for the two formulated problems: Starting from an empty set, we greedily and iteratively find and add a patient for isolation (or releasing) that leads to the smallest $\rho ({\varvec{A}})$ value until the size of the set reaches the given budget.

### Ethical statement

This project is approved by the IRB “Controlling healthcare associated infections: an in-silico framework” and meets the criteria of exempt research under 45CFR46.104(d)(4)iii. All methods were performed in accordance with the relevant guidelines and regulations. Ours was a retrospective study and hence informed consent was waived by the approving ethical committee.

## Results

In this section, we demonstrate the performance of our Greedy-Spectral algorithm for clinical problems using MRSA (a kind of HAI) as an example. Here, we use two time-varying heterogeneous contact networks constructed from clinical metadata (e.g., inpatient Data, doctor’s Notes, and MedAdmin Data) from the Epic-based SQL database at the University of Virginia (UVA) hospital to showcase our Greedy-Spectral algorithm could achieve robust good performance. One represents 294 days before COVID-19 (May 2019–Feb 2020), referred as the UVA-Precovid network, and another represents 294 days since the pandemic (May 2020–Feb 2021), referred as the UVA-Covid network. In these two networks, MRSA rates significantly declined in the UVA-Covid period, while networks became slightly denser, though many local properties (e.g., degree distribution, local clustering) were the same^54^. We first demonstrate the performance of Greedy-Spectral algorithm and several competing baselines in suppressing the MRSA outbreak for both high and relaxed precaution and cleaning scenarios (we will explain the two scenarios in later sections). We then investigate the patients picked by Greedy-Spectral using their electronic health records (EHRs) to see if they are meaningful from a clinical perspective.

**Figure 3:**
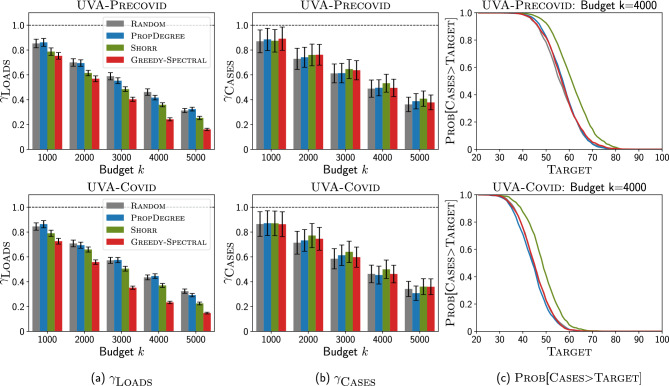
Greedy-Spectral achieves lower non-patient pathogen loads but similar number of new MRSA cases than other baselines under high precaution and cleaning scenario for isolation problem (IP). Top is for UVA-Precovid dataset, down is for UVA-Covid dataset. (**a**) Ratio of non-patient pathogen loads $\gamma _{\textsc {Loads}}$ (y-axis) with varying budget *k* (x-axis). Lower is better. The black bars show the standard error. (**b**) Ratio of new MRSA cases $\gamma _{\textsc {Cases}}$ (y-axis) with varying budget *k* (x-axis). Lower is better. (**c**) $\textsc {Prob}[\textsc {Cases}>\textsc {Target}]$ (y-axis) with varying Target (x-axis) for budget $k=4000$. Lower is better. See Supplementary Information for more experiment results.

### Performance for clinical problems

#### High precaution and cleaning scenario

We first use the parameterization by calibrating 2-Mode-SIS model to the weekly number of new tested positive MRSA cases collected from EHRs data for experiments (see Supplementary Information fore more details). This scenario corresponds to the normal level of precaution and cleaning practices in healthcare settings^55^, as in UVA, to avoid the outbreak of HAI including MRSA. We discuss the performance of Greedy-Spectral and other baselines under this scenario for the isolation problem (we show the results for clearance problem in Supplementary Information.)

**Figure 4:**
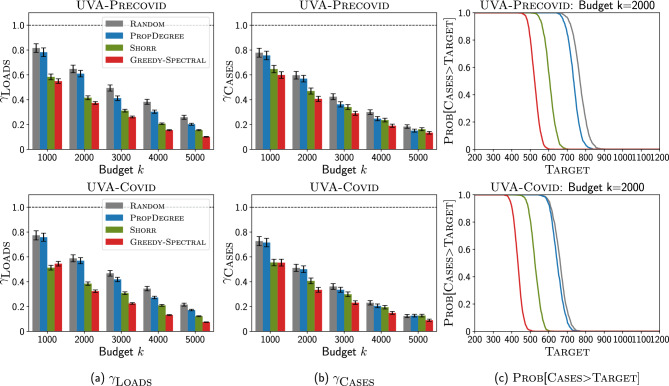
Greedy-Spectral achieves lower non-patient pathogen loads and number of new MRSA cases than other baselines under relaxed precaution and cleaning scenario for isolation problem (IP). Top is for UVA-Precovid dataset, down is for UVA-Covid dataset. (**a**) Ratio of non-patient pathogen loads $\gamma _{\textsc {Loads}}$ (y-axis) with varying budget *k* (x-axis). (**b**) Ratio of new MRSA cases $\gamma _{\textsc {Cases}}$ (y-axis) with varying budget *k* (x-axis). (**c**) $\textsc {Prob}[\textsc {Cases}>\textsc {Target}]$ (y-axis) with varying Target (x-axis) for budget $k=2000$. See Supplementary Information for more experiment results.

Figure 3 shows how Greedy-Spectral performs in minimizing non-patient pathogen loads and the number of new MRSA cases compared with other baselines: (1) Random will randomly pick *k* patients, (2) PropDegree will pick *k* patients with the probability of picking patients *i* proportional to its degree, (3) Shorr will pick *k* patients with the highest so-called Shorr scores^56^ (see Supplementary Information for more details). Note that Shorr was developed to assess the clinical risk of an admitted patient acquiring MRSA, better performance compared with Shorr means Greedy-Spectral is a more effective contact precaution strategy. As shown in Fig. 3a, Greedy-Spectral is more effective in reducing the amount of non-patient pathogen loads than other baselines. Here, $\gamma _{\textsc {Loads}}$ is defined as the ratio of non-patient (HCWs and location) pathogen loads against $k=0$ situation. For example, when $k=5000$, $\gamma _{\textsc {Loads}}$ for Greedy-Spectral is $\frac{\textsc {Loads}_{k=5000,\textsc {Greedy-Spectral}}}{\textsc {Loads}_{k=0}}$, where $\textsc {Loads}_{k=5000,\textsc {Greedy-Spectral}}$ and $\textsc {Loads}_{k=0}$ are the sum of HCWs pathogen loads and location pathogen loads when selecting 5000 patients for contact precaution using Greedy-Spectral, and when no patients are selected ($k=0$) respectively. In Fig. 3a, when $k=5000$, $\gamma _{\textsc {Loads}}$ for Greedy-Spectral is 9.2% (7.8%) lower than for Shorr on UVA-Precovid (UVA-Covid), this indicates that Greedy-Spectral is more effective in reducing the non-patient pathogen loads than other baselines (similar conclusion can also be drawn for other budget *k*). However, in Fig. 3b, we observe that our algorithm still gives a similar ratio of new MRSA cases $\gamma _{\textsc {Cases}}$, which is defined similarly as the ratio of the number of new MRSA cases against $k=0$ situation. In Fig. 3c, it also gives similar probability that the number of new MRSA cases is larger than Target, $\textsc {Prob}[\textsc {Cases}>\textsc {Target}]$ for varying $\textsc {Target}$ (x-axis) as other baselines. Here, lower loads achieved by Greedy-Spectral are not leading to fewer cases, which is likely because of the isolation practices currently in use.

#### Relaxed precaution and cleaning scenario

Next, we investigate how $\textsc {Greedy-Spectral}$ and other baselines perform in settings where precautions and cleaning practices are relaxed. Hospitals all over the world have already faced shortages induced by the COVID-19 pandemic, including personal protective equipment, nasal swabs for testing, laboratory diagnostic capacity, and medical personnel, e.g.,^57, 58^. Further, shortages in nursing staff have predated the COVID-19 pandemic, and is known to be associated with increased risk of HAI^59^. In the event of such shortages, it is hard to maintain the same level of precautions and cleaning policies, and the number of cases is likely to increase. Optimizing interventions becomes even more important in this setting. In this paper, we use the pathogen shedding rate $\alpha$ in the 2-Mode-SIS model to capture the influence of relaxed cleaning practices, and we use $\alpha$ leading to different numbers of cases when $k=0$ to capture different levels of relaxation of cleaning practices.

**Figure 5:**
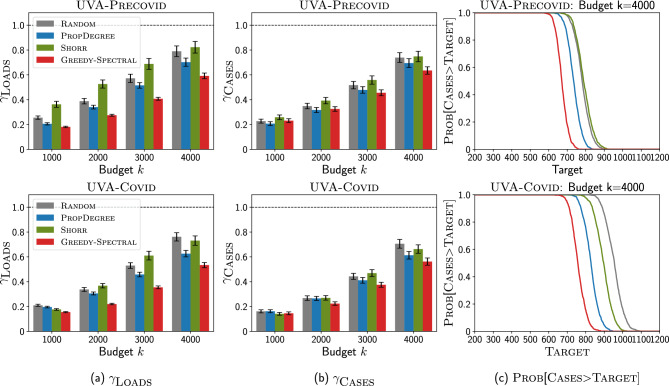
Greedy-Spectral achieves lower non-patient pathogen loads and number of new MRSA cases than other baselines under relaxed precaution and cleaning scenario for clearance problem (CP). Top is for UVA-Precovid dataset, down is for UVA-Covid dataset. (**a**) Ratio of non-patient pathogen loads $\gamma _{\textsc {Loads}}$ (y-axis) with varying releasing budget *k* (x-axis). (**b**) Ratio of new MRSA cases $\gamma _{\textsc {Cases}}$ (y-axis) with varying releasing budget *k* (x-axis). (**c**) $\textsc {Prob}[\textsc {Cases}>\textsc {Target}]$ (y-axis) with varying Target (x-axis) for releasing budget $k=4000$. See Supplementary Information for more experiment results.

*Isolation problem results* We first focus on isolation problem (IP) under relaxed precaution and cleaning scenario. Here, we demonstrate our Greedy-Spectral algorithm and other baselines by varying *k* but fixing $\alpha$ such that the number of cases is 1000 when $k=0$. We provide more results in Supplementary Information.

Fig. 4 shows the results with varying *k*. As shown in Fig. 4, Greedy-Spectral achieves lower non-patient pathogen loads and number of new MRSA cases than other baselines. In Fig. 4a, it achieves lower $\gamma _{\textsc {Loads}}$. For example, when $k=2000$, it leads to 4.2% (6.1%) lower loads than Shorr, and 23.6% (24.5%) lower loads than PropDegree on UVA-Precovid (UVA-Covid). Furthermore, our algorithm also leads to lower $\gamma _{\textsc {Cases}}$ as shown in Fig. 4b. When $k=2000$, it achieves 6.3% (7.2%) lower cases than Shorr, and 16.3% (16.6%) lower cases than PropDegree on UVA-Precovid (UVA-Covid). This indicates that our algorithm leads to around 60–70 fewer MRSA cases than Shorr and 160–170 fewer cases than PropDegree (since the number of cases is 1000 when $k=0$). In Fig. 4c, we show how $\textsc {Prob}[\textsc {Cases}>\textsc {Target}]$ changes with varying $\textsc {Target}$. Here, Greedy-Spectral always has lowest $\textsc {Prob}[\textsc {Cases}>\textsc {Target}]$. For example, for UVA-Precovid (top row) and $\textsc {Target}=600$, $\textsc {Prob}[\textsc {Cases}>\textsc {Target}]$ for our algorithm is 0, indicating that it can always lead to less than 600 cases. However, Shorr has 56.1% probability of having more than 600 cases, and PropDegree and Random have 100% probability of having more than 600 cases. This demonstrates the effectiveness of Greedy-Spectral algorithm in leading to fewer cases.

*Clearance problem results* We repeat the experiments shown for isolation problem under relaxed precaution and cleaning scenario above to clearance problem. Similarly, we demonstrate our Greedy-Spectral algorithm and other baselines by varying *k* but fixing $\alpha$ such that the number of cases is 1000 when $k=0$. We also provide more results in Supplementary Information.

Figure 5 shows the results with varying *k*. As shown in Fig. 5, Greedy-Spectral achieves lower non-patient pathogen loads and number of new MRSA cases than other baselines for the clearance problem. In Fig. 5a, it achieves lower non-patient pathogen loads. Furthermore, it also leads to a lower number of new MRSA cases as shown in Fig. 5b. For example, when $k=4000$ (i.e. releasing 4000 patients) for UVA-Covid (bottom row), our algorithm leads to 8.5% lower loads and 6.9% lower $\gamma _{\textsc {Cases}}$ than other baselines. Besides, it also has lowest $\textsc {Prob}[\textsc {Cases}>\textsc {Target}]$ in Fig. 5c. For example for UVA-Precovid (top row), when releasing $k=4000$ patients and $\textsc {Target}=700$, Greedy-Spectral has only 12.3% probability of having more than 700 cases. Instead, other baselines have 100% probability of having more than 700 cases.

**Figure 6:**
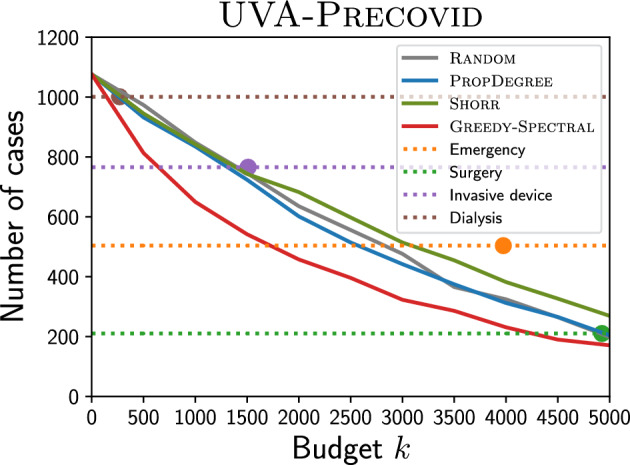
Comparison against clinical heuristic precaution policies. The x-axis is budget *k*, the y-axis is the number of cases. The horizontal dashed lines represent the simulated number of MRSA cases with the these policies, and scattered dots correspond to the number of patients isolated by these policies.

#### Comparison with clinical heuristic precaution policies

Many clinical heuristic precaution policies are also used in practice^60, 61^. Usually, these policies tend to pick patients with high MRSA risks. For example, patients with surgery history or invasive device use may be considered as having high risk and may be picked for precaution. We next leverage EHRs to implement these clinical heuristic policies and compare them with our Greedy-Spectral algorithm. The EHRs of patients in the UVA dataset include information like surgery history or underlying diseases (for brevity, we call them ‘features’) for each patient. Specifically, we compare with the following four heuristic policies: isolate all emergency patients, isolate all patients with surgery history within 90 days, isolate all patients with invasive device use history within 90 days, and isolate all patients with dialysis history within 90 days. The four features used in these policies are all considered to be highly risky^56, 60, 61^. As shown in Fig. 6 for UVA-Precovid, Greedy-Spectral can also suppress MRSA outbreak better than these clinical heuristic precaution policies. Here, horizontal dashed lines represent the simulated number of new cases for these four policies. Scattered dots correspond to the number of patients isolated by each policy. We can see the line for our algorithm is below all scattered dots. This indicates that when isolating the same number of patients, Greedy-Spectral leads to fewer MRSA cases than these policies.

### Case study: analysis on isolated patients

We also investigate the patients picked by our Greedy-Spectral algorithm from a clinical perspective. In Table 1, we use PropDegree, Shorr, and Greedy-Spectral to pick 500 patients for precaution for UVA-Precovid, and then show the fraction of patients with highly risky features^60, 61^ (note that one patient may have multiple features and can be counted multiple times). We also list the fraction of patients with these features in population for comparison. As shown in Table 1, our algorithm Greedy-Spectral picks meaningful patients with these features for precaution. For example, compared with PropDegree and fraction in population, it picks many more patients with invasive device use history and dialysis history. Compared with Shorr, Greedy-Spectral highlights patients having surgery within 90 days more while emphasizing dialysis patients less. Additionally, Greedy-Spectral also captures the structural information of the heterogeneous contact networks well since the average degree of the patients picked by it is larger than other baselines. This explains why it performs better than Shorr in suppressing MRSA outbreaks. *Note that our algorithm uses only network information, and not patient level feature information, but it still identifies the correct patients at high risk and performs better in minimizing the MRSA spread as shown above*.

**Table 1:** Fraction of patients with different features.

Features	Population	$\textsc {PropDegree}$	$\textsc {Shorr}$	$\textsc {Greedy-Spectral}$
Surgery (within 90 days)	0.601	0.614	0.696	0.750
Surgery (90+ days)	0.377	0.434	0.572	0.572
Invasive Device (within 90 days)	0.233	0.264	0.472	0.470
Invasive Device (90+ days)	0.171	0.180	0.356	0.382
Dialysis (within 90 days)	0.041	0.050	0.184	0.136
Dialysis (90+ days)	0.031	0.042	0.160	0.096
ICU	0.281	0.326	0.508	0.402

In Table 2, we also investigate the differences in the type of patients picked in the UVA-Precovid and UVA-Covid period. Here, we list the fraction of patients with highly risky features picked by Greedy-Spectral in both periods. As shown in Table 2, our algorithm is picking 13.2% more patients with invasive device use history within 90 days, 20.6% more patients with dialysis history within 90 days and 19.4% more patients in ICU in $\textsc {UVA-Covid}$ than $\textsc {UVA-Precovid}$. For example, the increasing number of patients picked in ICU by our algorithm can be explained by the fact that ICU is getting more crowded. According to the EHRs data, there are more contacts in the UVA-Covid period than in the UVA-Precovid period (average degree of patients in ICU: 115.33 (UVA-Precovid) va. 125.24 (UVA-Covid)). Additionally, the average degree of the patients picked by our algorithm for UVA-Covid period is also larger than UVA-Precovid.

**Table 2:** Fraction of patients with different features for $\textsc {Greedy-Spectral}$ on $\textsc {UVA-Precovid}$ and $\textsc {UVA-Covid}$ dataset.

Notations	$\textsc {UVA-Precovid}$	$\textsc {UVA-Covid}$
Surgery (within 90 days)	0.750	0.752
Surgery (90+ days)	0.572	0.6
Invasive device (within 90 days)	0.470	0.532
Invasive device (90+ days)	0.382	0.342
Dialysis (within 90 days)	0.136	0.164
Dialysis (90+ days)	0.096	0.086
ICU	0.402	0.48

## Discussion

We develop a spectral characterization for 2-Mode-SIS^34^ for modeling the spread of HAIs, such as MRSA pathogens, which captures the long-term dynamics of pathogen spread by taking into account both the contact network structure and disease parameters. This model has been proposed as an alternative to the standard SIS models, but has not been studied rigorously before. We also study two important clinical problems arising from control of HAIs, and show how these can be formalized using our spectral characterization. We design Greedy-Spectral, an algorithm for solving these clinical problems, which greedily and iteratively finds and adds a patient to isolate (or relase) until reaching the budget.

Overall, our results demonstrate that our spectral characterization and Greedy-Spectral algorithm hold sufficient potential for minimizing the spread of MRSA. In experiments, we compare Greedy-Spectral with several baselines including Shorr, which picks patients for contact precautions using their features collected from Electronic Health Records. For the high precaution and cleaning scenario, which corresponds to the normal level of pathogens and cleaning practices in healthcare settings, our simulations suggest that the Greedy-Spectral algorithm outperforms the Shorr by achieving lower non-patient pathogen loads. For the relaxed precaution and cleaning scenario, which captures the shortage of resources (e.g., personal protective equipment, laboratory diagnostic capacity, and medical personnel in the COVID-19 pandemic^57, 58^), our simulations indicate that Greedy-Spectral algorithm not only reduces pathogen loads but also leads to fewer MRSA cases compared with Shorr. Additionally, our algorithm uses only the network information and not the patient-level feature information. However, it still identifies the correct patients at high risk and performs better in minimizing the MRSA spread as shown above.

Our Greedy-Spectral algorithm is likely to be helpful in controlling MRSA and other healthcare-associated infections. As demonstrated in our simulations, our algorithm uses only the network information rather than patient-level features, yet it successfully identifies high-risk patients and outperforms Shorr and other baselines in minimizing MRSA spread. In practice, the Greedy-Spectral algorithm can be implemented by constructing contact networks using EHR data such as inpatient data, doctors’ notes, and MedAdmin data, which are as similarly available as patient-level features. Some research also suggests that wearable RFID sensors can be used to construct networks^62, 63^. With the contact networks, people can then run our Greedy-Spectral algorithm to decide the most suitable patients to put under or released from precaution (corresponding to the isolation problem and clearance probelm respectively). We expect our approach will assist epidemiologists and clinicians in identifying appropriate patients for contact precautions, ultimately aiding in the control of MRSA transmission. Our spectral characterization and Greedy-Spectral algorithm can also be extended to other diseases and epidemiological models.

One of the limitations of this work is that the 2-Mode-SIS model we use involves only two states: susceptible and infected. Real-world MRSA infections, however, manifest in various forms (e.g., bloodstream infections and pneumonia)^64^, while the 2-Mode-SIS model simplifies them into a single infected state. In addition, the 2-Mode-SIS model assumes that any susceptible patient can be colonized instead of explicitly incorporating a colonization state in it itself. One potential extension of this work could extend our spectral characterization analysis to more complex models that could distinguish different kinds of MRSA infections and colonization. Our approach can also be adapted for other healthcare-associated infections, such as *C. difficile*, by substituting the 2-Mode-SIS model with other models. Another limitation is that we assume that patients selected for contact precautions remain isolated throughout the entire period. However, in actual clinical practice, patients may be released from contact precautions when they recover or are discharged from the hospital. Future work could consider the possibility of isolating or releasing patients at any time step to gain more flexibility. Although contact networks and other parts of EHR data are very sensitive datasets, the privacy concerns can be mitigated by running our Greedy-Spectral algorithm on anonymous contact networks to estimate the impact of the optimal solution, and use characteristics of the solution in defining actual strategies.

### Supplementary Information

Supplementary Information 1.

## Data Availability

The simulation outputs of our model are available in 10.5281/zenodo.7811397. The electronic health record (EHR) data used in developing the models is not available since it is highly sensitive, and we do not have permission to release it.

## Code availability

Code have been deposited in 10.5281/zenodo.7811397.
