# Supplementary material for: Using spectral characterization to identify healthcare-associated infection (HAI) patients for clinical contact precaution
Source: Sci Rep. 2023 Sep 27;13:16197. doi: 10.1038/s41598-023-41852-5 (PMC10533902; doi:10.1038/s41598-023-41852-5)
Supplement: Supplementary file 1 — Supplementary Information 1. [file 41598_2023_41852_MOESM1_ESM.pdf]

Supplementary Information

Related works

Due to the significant health burden of HAIs[1–6], epidemiologists and clinicians have proposed many approaches to control the HAI spread in hospital: These methods include contact tracing[7], antimicrobial stewardship[8], and contact precautions[9]. Contact tracing is to identify the patients who have been in close contact with a person diagnosed with HAIs[7]. They have been shown to be effective in controlling in-hospital COVID-19 outbreaks[10]. Antimicrobial stewardship is to manage the use of antibiotics to avoid overuse and misuse. They have been shown to be effective in reducing the number of HAI cases[11]. However, decreased antibiotic use could lead to a substantial increase in patient risk[12]. Contact precautions implement safety procedures, such as wearing gloves and gowns, to prevent the spread of infections that can be transmitted through direct or indirect pathways. Compared with other HAI control methods, contact precautions are more effective in preventing further nosocomial infections and easier to implement, and therefore have been widely used in most hospitals[9,13].

However, as described in the main article, the restricted contact precaution budget requires we can only apply them to a small fraction of patients, which leads to two clinical problems (isolation problem (IP) and clearance problem (CP)). For the isolation problem, existing works such as selecting top-k influential nodes in networks [14–18] have shown good performance in preventing many other diseases[19]. However, our isolation problem is more complex because of the complex spread dynamics of HAI, which makes these existing algorithms less reliable. For the clearance problem, although there are some clinical guidelines on removing patients from contact precautions[20,21]. However, these guidelines are restricted to patient-level decisions while ignoring their role in HAI spread at the hospital level.

In this work, we use spectral characterization to help formulate the two clinical problems and propose our GREEDY-SPECTRAL algorithm to solve them. Such methods have been widely used to find the epidemic threshold on graphs to determine when an epidemic is expected to die out[22–24]. However, most of them are limited to direct person-to-person contact networks. To the best of our knowledge, our work is the first to apply such spectral characterization to such complex models on heterogeneous contact networks.

Heterogeneous contact networks

$\mathscr{G}$ is a set of time-varying heterogeneous contact networks - it is sequence of graphs $\mathscr{G}_1, \mathscr{G}_2, \ldots, \mathscr{G}_T$ with T number of timesteps. We focus on unweighted and undirected graphs; hence each $\mathscr{G}_t$ is represented as an $N \times N$ symmetric binary adjacency matrix $\boldsymbol{A}_t$ s.t. $\boldsymbol{A}_t(i,j) = 1$ if nodes i and j are connected with an edge at time t, $\boldsymbol{A}_t(i,j) = 0$ otherwise. Note that our node-set is shared across all graphs and can represent people (patients and HCWs) as well as locations (patient rooms, nurses' stations, etc.).

As mentioned earlier, contact networks are not explicitly available in EHR data. Instead, we infer them from various tables, such as Flowsheets, Medicine Administration, Admission Discharge, and doctor's notes. These tables specify specific events that occurred during the patient's stay in the hospital, e.g., admission into the hospital or ICUs, administration of different kinds of medications. One can infer the exact location of the patient, and when a HCW enters the patient room, which we use to infer co-location. The contact network is constructed using this co-location information.

2-MODE-SIS model

In this work, we use 2-MODE-SIS model[25] to simulate the spread of MRSA pathogens. Procedure 1 shows the simulation steps of the model. The meaning of notations and parameters are listed in Supplementary Table 1 and Supplementary Table 2.

Supplementary Table 1. *List of notations*

Notation	Description
P	Number of patients
H	Number of Healthcare workers (HCWs)
L	Number of locations
N	Total number of nodes ($N = P + H + L$)
M	Total number of edges
T	Number of timesteps
$\boldsymbol{l}_t$	Pathogen load vector at time t
$\boldsymbol{x}_t$	Infection state vector at time t
$\boldsymbol{p}_t$	Infection probability vector at time t ($\boldsymbol{p}_t = \mathbb{E}[\boldsymbol{x}_t]$)
$\boldsymbol{A}_t$	Adjacency matrix at time t
$\boldsymbol{R}_t$	Pathogen transfer matrix at time t
$\rho(\boldsymbol{M})$	Spectral radius (i.e., the largest eigenvalue) of matrix $\boldsymbol{M}$
$\text{nnz}(\boldsymbol{M})$	Number of nonzeros in matrix $\boldsymbol{M}$

Supplementary Table 2. *List of* 2-MODE-SIS *model parameters*

Notation	Description
α	Pathogen shedding rate
β	Disease infectivity
δ	Recovery probability
ϕ_i	Natural pathogen reduction rate on node i
τ_{ijt}	Transfer ratio from node j to node i at time t

In the contact networks, each node carries some amount of pathogens that changes over time, and the exchange of pathogens among the nodes is driven by edges that imply close contact among the nodes. 2-MODE-SIS model uses τ_{ijt} to denote the ratio of pathogen being transferred (or remaining if $i = j$) from node j to node i at time t, and ϕ_i to denote the natural pathogen reduction rate on node i. By constructing a transfer matrix $\boldsymbol{R}_t$ from the transfer ratios τ_{ijt}, reduction rates ϕ_i, and adjacency matrix $\boldsymbol{A}_t$, the pathogen load updates can be written as a linear operation as in Procedure 1 step 5, where $\boldsymbol{x}_t$ is the infection state vector at time t and α is pathogen shedding rate for infected patients. Note that we restrict the column-sums of $\boldsymbol{R}_t$ to be less than or equal to 1, which implies that the total amount of pathogen cannot increase after transfer (i.e., $\|\boldsymbol{R}_t\boldsymbol{l}_t\|_1 \leq \|\boldsymbol{l}_t\|_1$).

2-MODE-SIS is pathogen 'load-based' where each patient can be either *susceptible* or *infected*, and the model keeps track of pathogen load on all people and locations. Such pathogen load based models are widely used in HAIs modeling [7,26–31]. Besides, they can also model the cleaning practice widely used in contact precautions (e.g., using disinfectants to clean the ward) directly[26,29,30,32,33]. Therefore, compared with traditional Independent Cascade (IC)/Susceptible-Infectious-Susceptible(SIS) models, 2-MODE-SIS model is more suitable for HAIs modeling. Unlike classical SIS models[34], in 2-MODE-SIS model the infection probability of a patient increases with the amount of pathogen on the patient, which is formulated as a dose-response function $f(\cdot)$ in Procedure 1 step 8. Once infected, the patient sheds α additional pathogen per timestep to his own load, which can later be transferred to neighbors (both people and locations) via edges; this shedding continues until the patient recovers. For susceptible patients, they may still be colonized with the pathogen loads and spread them to others. As for HCWs and locations that are assumed to be non-infectable, they still act as pathways of pathogen transfer.

Procedure 1 2-MODE-SIS model

1: **Inputs:** $\Theta = \left\{\alpha, \beta, \delta, \{\boldsymbol{A}_t\}_{t=1}^T, \{\tau_{ijt}\}_{i,j,t}\right\}$
2: Initialize infection-states $\boldsymbol{x}_1$ and loads $\boldsymbol{l}_1$
3: Compute $\boldsymbol{R}_t(i,j) = \begin{cases} \tau_{ijt}\boldsymbol{A}_t(i,j) & \text{if } i \neq j \\ \tau_{ijt}\phi_i & \text{if } i = j \end{cases}$ for all t.
4: **for** $t = 1, \ldots, T$ **do**
5:     Update loads $\boldsymbol{l}_{t+1} = \boldsymbol{R}_t\boldsymbol{l}_t + \alpha\boldsymbol{x}_t$.
6:     **for** each patient i **do**
7:         **if** i is susceptible at time t (i.e., $\boldsymbol{x}_t(i) = 0$) **then**
8:             i gets infected (i.e. $\boldsymbol{x}_{t+1}(i) = 1$) with probability $f(\boldsymbol{l}_t(i)) = \min\{1, \beta\boldsymbol{l}_t(i)\}$.
9:         **else**
10:             i gets susceptible (i.e. $\boldsymbol{x}_{t+1}(i) = 0$) with probability δ.
11:         **end if**
12:     **end for**
13: **end for**

Approximation via NLDS

In this work, we develop a characterization that captures the overall dynamics of the system and derive a spectral characterization for the 2-MODE-SIS model. We will also show in later sections how it helps formulate the clinical problems.

Our proposed approach is to approximate the propagation dynamics of the 2-MODE-SIS model using a Non-Linear Dynamical System (NLDS) with two coupled states: the pathogen loads $\boldsymbol{l}_t$ and the infection probabilities $\boldsymbol{p}_t$. For simplicity, assume that the system periodically operates on two pathogen transfer matrices, $\boldsymbol{R}_1$ for odd timesteps and $\boldsymbol{R}_2$ for even timesteps. Hence, the time-varying contact network will be $\{\boldsymbol{R}_1, \boldsymbol{R}_2, \boldsymbol{R}_1, \boldsymbol{R}_2, \cdots, \boldsymbol{R}_1, \boldsymbol{R}_2\}$. Note that the following analysis can easily be generalized toward any arbitrary number of repeating pathogen transfer matrices. Given this assumption, the state transition

updates for odd timesteps are

$$\boldsymbol{l}_{2t+1} = \boldsymbol{R}_1 \boldsymbol{l}_{2t} + \alpha \boldsymbol{x}_{2t} \tag{1}$$

$$\boldsymbol{x}_{2t+1}(i) = \mathbb{1}_{\text{cure}}^{(i,2t)} \boldsymbol{x}_{2t}(i) + \mathbb{1}_{\text{load}}^{(i,2t)} (1 - \boldsymbol{x}_{2t}(i)) \tag{2}$$

where $\mathbb{1}_{\text{cure}}^{(i,2t)}$ and $\mathbb{1}_{\text{load}}^{(i,2t)}$ are indicator variables of the event at time $2t$ that an infected patient i is cured (with probability δ) and the event where patient i becomes infected (with probability $\beta \boldsymbol{l}_{2t}(i)$), respectively. In Equation 2, the first term vanishes when i is susceptible, and if i is infected instead, the second term vanishes.

From linearity of expectation, we get:

$$\mathbb{E}[\boldsymbol{l}_{2t+1}] = \boldsymbol{R}_1 \mathbb{E}[\boldsymbol{l}_{2t}] + \alpha \boldsymbol{p}_{2t} \tag{3}$$

$$\mathbb{E}[\boldsymbol{x}_{2t+1}(i)] = \mathbb{E}[\mathbb{1}_{\text{cure}}^{(i,2t)} \boldsymbol{x}_{2t}(i)] + \mathbb{E}[\mathbb{1}_{\text{load}}^{(i,2t)} (1 - \boldsymbol{x}_{2t}(i))]$$

$$\Rightarrow \boldsymbol{p}_{2t+1}(i) = (1-\delta)\boldsymbol{p}_{2t}(i) + \beta \boldsymbol{l}_{2t}(i)(1 - \boldsymbol{p}_{2t}(i)) \tag{4}$$

Given the current state at time $2t$, it is reasonable to assume that $\mathbb{1}_{\text{load}}^{(i,2t)}$ and $\boldsymbol{x}_{2t}(i)$ are independent as the two random variables are correlated indirectly through the past infection states. This allows us to use $\mathbb{E}[\mathbb{1}_{\text{load}}^{(i,2t)}(1 - \boldsymbol{x}_{2t}(i))] = \mathbb{E}[\mathbb{1}_{\text{load}}^{(i,2t)}]\mathbb{E}[1 - \boldsymbol{x}_{2t}(i)]$. As a sidenote, this independence holds when patient i has been susceptible at time t' for all $t' < 2t$.

Let $\boldsymbol{s}_{2t} = \begin{bmatrix} \boldsymbol{p}_{2t} \\ \boldsymbol{l}_{2t} \end{bmatrix} \in \mathbb{R}^{(P+N)}$ be the vector describing the state of the system at time $2t$. Combining Equations 3 and 4 allows us to write the transition at odd timesteps as

$$\boldsymbol{s}_{2t+1} = g_1(\boldsymbol{s}_{2t}) := \begin{bmatrix} (1-\delta)\boldsymbol{p}_{2t}(i) + \beta \boldsymbol{l}_{2t}(i)(1 - \boldsymbol{p}_{2t}(i)) \\ \boldsymbol{R}_1 \boldsymbol{l}_{2t} + \alpha \boldsymbol{p}_{2t} \end{bmatrix}$$

where g_1 is a non-linear mapping. Similarly, for even timesteps, we get (with non-linear mapping g_2)

$$\boldsymbol{s}_{2t+2} = g_2(\boldsymbol{s}_{2t+1}) := \begin{bmatrix} (1-\delta)\boldsymbol{p}_{2t+1}(i) + \beta \boldsymbol{l}_{2t+1}(i)(1 - \boldsymbol{p}_{2t+1}(i)) \\ \boldsymbol{R}_2 \boldsymbol{l}_{2t+1} + \alpha \boldsymbol{p}_{2t+1} \end{bmatrix}$$

Stability analysis of the NLDS

Equipped with the non-linear equations of our approximated NLDS, we can then analyze its asymptotic behavior. The long-term behavior of a dynamical system is dictated by the stability of its equilibrium points, at which the states no longer change over time (i.e., $\boldsymbol{s}^{eq} = g(\boldsymbol{s}^{eq})$). The particular equilibrium point of interest is the *all-zero* point $\mathbf{0}$ where there is no infection as well as no pathogen remaining. Clearly, both g_1 and g_2 map $\mathbf{0}$ onto itself and $\mathbf{0}$ is an equilibrium point. We aim to find under what condition this equilibrium point is 'stable'[24] (i.e. a small perturbation does not cause large deviations). We use the next claim on asymptotic stability in a discrete-time NLDS.

Lemma 1. *Assume a NLDS with discrete-time updates $\boldsymbol{s}_{t+1} = g(\boldsymbol{s}_t)$ has an equilibrium point at $\boldsymbol{s}^{eq}$. Then, the point $\boldsymbol{s}^{eq}$ is asymptotically stable if the spectral radius (i.e., the largest eigenvalue) of the Jacobian matrix $\boldsymbol{J} = \nabla g = \left[\frac{\partial g_i}{\partial \boldsymbol{s}_t(j)}\right]_{ij}$ evaluated at $\boldsymbol{s}^{eq}$ is less than 1[35].*

Specifically, in the system with two update steps g_1 and g_2, each of which the Jacobian matrices evaluated at $\mathbf{0}$ are

$$\nabla g_1|_{\boldsymbol{s}_{2t}=\mathbf{0}} = \left.\frac{\partial \boldsymbol{s}_{2t+1}}{\partial \boldsymbol{s}_{2t}}\right|_{\boldsymbol{s}_{2t}=\mathbf{0}} = \begin{bmatrix} (1-\delta)\boldsymbol{I}_P & \beta \boldsymbol{I}_P \quad \mathbf{0} \\ \alpha \boldsymbol{I}_P & \\ \mathbf{0} & \boldsymbol{R}_1 \end{bmatrix} =: \boldsymbol{S}_1$$

$$\nabla g_2|_{\boldsymbol{s}_{2t+1}=\mathbf{0}} = \left.\frac{\partial \boldsymbol{s}_{2t+2}}{\partial \boldsymbol{s}_{2t+1}}\right|_{\boldsymbol{s}_{2t+1}=\mathbf{0}} = \begin{bmatrix} (1-\delta)\boldsymbol{I}_P & \beta \boldsymbol{I}_P \quad \mathbf{0} \\ \alpha \boldsymbol{I}_P & \\ \mathbf{0} & \boldsymbol{R}_2 \end{bmatrix} =: \boldsymbol{S}_2$$

The following lemma shows that the long-term behavior can be characterized by computing the spectral radius of a so-called *system matrix*, defined as the product of individual Jacobian matrices $\boldsymbol{S}_2$ and $\boldsymbol{S}_1$. This result is derived by viewing the system as $g(\boldsymbol{s}_{2t}) := g_2(g_1(\boldsymbol{s}_{2t}))$ and applying the chain rule to its Jacobian.

Lemma 2. *If the spectral radius (i.e., the largest eigenvalue) of the system matrix $\boldsymbol{S} = \boldsymbol{S}_2\boldsymbol{S}_1$ is less than 1 (i.e. $\rho(\boldsymbol{S}) < 1$), then the all-zero equillibrium of the NLDS is asymptotically stable.*

Proof. Consider another system that uses the same update rules, but takes two timesteps at once. This system can be written as $\boldsymbol{s}_{2t+2} = g(\boldsymbol{s}_{2t})$ where $g(\boldsymbol{s}_{2t}) := g_2(g_1(\boldsymbol{s}_{2t}))$. These two systems are equivalent, and the Jacobian of g evaluated at $\mathbf{0}$ can be written as a product two Jacobians evaluated on the same point.

$$\begin{aligned}\left.\frac{\partial \boldsymbol{s}_{2t+2}}{\partial \boldsymbol{s}_{2t}}\right|_{\boldsymbol{s}_{2t}=\mathbf{0}} &= \left.\left(\frac{\partial \boldsymbol{s}_{2t+2}}{\partial \boldsymbol{s}_{2t+1}} \times \frac{\partial \boldsymbol{s}_{2t+1}}{\partial \boldsymbol{s}_{2t}}\right)\right|_{\boldsymbol{s}_{2t}=\mathbf{0}} \\ &= \left.\frac{\partial \boldsymbol{s}_{2t+2}}{\partial \boldsymbol{s}_{2t+1}}\right|_{\boldsymbol{s}_{2t+1}=\mathbf{0}} \times \left.\frac{\partial \boldsymbol{s}_{2t+1}}{\partial \boldsymbol{s}_{2t}}\right|_{\boldsymbol{s}_{2t}=\mathbf{0}} \\ &= \boldsymbol{S}_2\boldsymbol{S}_1 \end{aligned} \tag{5}$$

The first equality is due to chain rule, and the second comes from the fact that $\mathbf{0}$ is an equilibrium point (i.e., $\boldsymbol{s}_{2t} = \mathbf{0}$ implies $\boldsymbol{s}_{2t+1} = \mathbf{0}$). Then, applying Lemma 1 to Equation 5 completes the proof. □

As mentioned before, this argument can be generalized to any arbitrary number of repeating pathogen transfer matrices (i.e, from only repeating $\boldsymbol{R}_1$ and $\boldsymbol{R}_2$ to repeating $\boldsymbol{R}_1, \boldsymbol{R}_2, \cdots, \boldsymbol{R}_T$), which leads to our main result.

Theorem 1 (Spectral characterization)**.** *If the spectral radius (i.e., the largest eigenvalue) of the system matrix* $\boldsymbol{S} = \prod_{t=1}^{T} \boldsymbol{S}_{T-t+1}$ *is less than 1 (i.e.* $\rho(\boldsymbol{S}) < 1$*), then the all-zero equillibrium of the NLDS is asymptotically stable.*

Here we show an example simulation results on the LYON hospital dataset with synthetic parameters to demonstrate our characterization. We vary $\rho(\boldsymbol{S})$ with the shedding-rate α. Supplementary Fig 1(a) shows the varying infection trajectories as well as the corresponding $\rho(\boldsymbol{S})$ values. When $\rho(\boldsymbol{S}) > 1$, the system reaches an endemic state with nonzero infections, whereas when $\rho(\boldsymbol{S}) < 1$, we see fast decline towards the zero-infection state. Supplementary Fig 1(b) displays the threshold on $\rho(\boldsymbol{S})$ at which the dynamics of the system change. The endemic state footprint, measured as the average infected fraction across the last 50 timesteps, stays at 0 until $\rho(\boldsymbol{S})$ reaches 1 as expected from Theorem 1. As soon as $\rho(\boldsymbol{S})$ exceeds 1, the footprint suddenly takes off to a nonzero infection fraction, indicating a shift from gradual extinction to a long-lasting epidemic. Although this $\rho(\boldsymbol{S})$ is different from the basic reproductive number R_0 for the 2-MODE-SIS model since the computation of $\rho(\boldsymbol{S})$ also takes the pathogen loads $\boldsymbol{l}_t$ into consideration. However, they are highly related and share the same tipping point ($\rho(\boldsymbol{S}) = 1$ vs. $R_0 = 1$).

Explaining counter-intuitive behaviors

We also show that our characterization correctly captures and helps explain counter-intuitive behaviors of pathogen load-based propagation which have been observed before, specifically the so-called 'dilution effect'[25,36,37]. In Supplementary Fig 2. we show the simulation results on static Erdős-Rényi random graphs with increasing link probability p. The transfer ratios τ_{ijt} for $i \neq j$ and ϕ_i are fixed, such that the only difference between the curves are the number of edges in the graph and the proportion of pathogen remaining in each node after transfer (τ_{ijt} for $i = j$). As p increases, the number of edges are also increasing in this situation. However, we observe that the number of infections at the end are decreasing with increasing p. This is surprising, because this goes against our intuition that more contacts lead to more infections. This also contrasts with previous Independent Cascade/Linear Threshold/SIR style models where adding an edge leads to more infections[38]. However, our $\rho(\boldsymbol{S})$ correctly captures this dynamics. The reason is that with more edges in the graph, more pathogen is sent out and less remains, which means lower τ_{ijt} for $i = j$. Hence, the diagonal elements of $\boldsymbol{S}$ decreases, and $\rho(\boldsymbol{S})$ also decreases.

Applications of $\rho(\boldsymbol{S})$ for clinical problems

Using $\rho(\boldsymbol{S})$ to formalize clinical problems

Here, we show how our spectral characterization $\rho(\boldsymbol{S})$ can help formulate the two clinical problems IP and CP. The underlying intuition is that our spectral characterization well captures the transition dynamics of the HAIs breakout, and a lower $\rho(\boldsymbol{S})$ value should correspond to fewer infections. We first formalize the IP problem as follows:

Formal Isolation Problem (FIP). *Let* $\boldsymbol{S}(\Theta, P)$ *denote the system matrix given input parameters* Θ *and patients in set* P *under precaution. Given all patients* X *and a budget constraint* k*, find the subset of patients* P^* *for precaution s.t.*

$$P^* = \arg\min_{P} \rho(\boldsymbol{S}(\Theta, P)) \text{ subject to } P^* \subset X \text{ and } |P^*| = k$$

.

This formulation uses $\rho(\boldsymbol{S}(\Theta, P))$ to capture how isolating patients in P will affect the epidemic. Here, we formally model bringing a patient i under precaution by zeroing out off-diagonal terms in the i-th row and column in each pathogen transfer

matrix $\boldsymbol{R}_t$ (i.e., cut off its links with other nodes), and reducing the diagonal terms $\boldsymbol{R}_t(i,i)$ by a multiplicative factor $\phi_{ppe} < 1$ (i.e., reduce its remaining pathogen via cleaning). They are widely used together in hospitals for contact precautions[9,21,39]. Note that the current setup can be readily modified to capture other contact precaution practices while maintaining a similar problem formulation. Similarly, we can also formalize the CP problem:

Formal Clearance Problem (FCP). *Let $\boldsymbol{S}(\cdot)$ be defined as in FIP. Given an initial set Q of d candidate patients already under precaution ($|Q| = d$) and a budget constraint $k < d$, find the subset of patients P^* for clearance s.t.*

$$P^* = \arg\min_{P} \rho(\boldsymbol{S}(\Theta, Q \setminus P)) \text{ subject to } P \subset Q \text{ and } |P| = k$$

Algorithms

Unfortunately, both formal problems are NP-hard. This can be seen via a reduction from the well-known Independent Set problem[40] or a more similar Nodal Spectral Radius Minimization (NSRM) problem[41], both known to be NP-hard. For brevity, we defer detailed proofs later. However, computational hardness is not the only challenge. The optimization process itself can also incur numerical issues due to finite precision. We first discuss this issue in detail and develop practical algorithms for FIP and FCP via surrogate goals, which sidestep this issue.

Numerical issue

A natural algorithm for FIP or FCP would try adding (or removing) each candidate patient P under (or from) precaution and then compute by how much $\rho(\boldsymbol{S})$ changes to incrementally build a feasible solution set while minimizing $\rho(\boldsymbol{S})$. In practice, however, we find that computing the change in $\rho(\boldsymbol{S})$ due to a single node removal runs into numerical issues: If there are much more patient-to-non-patient interactions than patient-to-patient interactions, the change in $\rho(\boldsymbol{S})$ w.r.t. P can be too small to be computed within finite precision. The issue even gets worse when the total number of nodes N and the number of timesteps T increase.

Justification: To see why, let us decompose the pathogen transfer matrix $\boldsymbol{R}_t$ in $\boldsymbol{S}_t$ into rows corresponding to patients (who can be infected) and rows corresponding to non-patients (who cannot be infected). Then the $\boldsymbol{R}_{t,pp}$ submatrix of $\boldsymbol{R}_t$ encodes the patient-to-patient interactions at time t, $\boldsymbol{R}_{t,ph}$ encodes the patient-to-non-patient interactions at time t, and so on. Note that when entries in $\boldsymbol{R}_t$ are much smaller than $(1-\delta)$ and α (as in real parameter settings), the spectrum of $\boldsymbol{S}_t$ is mostly governed by the upper-left submatrix of $\boldsymbol{S}_t$ (enclosed in red square).

$$\boldsymbol{S}_t = \begin{bmatrix} (1-\delta)\boldsymbol{I}_p & \beta\boldsymbol{I}_p & \boldsymbol{0} \\ \alpha\boldsymbol{I}_p & \boldsymbol{R}_{t,pp} & \boldsymbol{R}_{t,ph} \\ \boldsymbol{0} & \boldsymbol{R}_{t,hp} & \boldsymbol{R}_{t,hh} \end{bmatrix}$$

This can be seen by viewing $\boldsymbol{R}_{t,ph}$ and $\boldsymbol{R}_{t,hp}$ as small perturbations added to a block-diagonal matrix of which the eigenvalues equal those of the upper-left submatrix in red, and $\boldsymbol{R}_{t,hh}$. Hence if the majority of interactions are between patients and non-patients rather than among patients, most changes will occur in $\boldsymbol{R}_{t,ph}$ and $\boldsymbol{R}_{t,hp}$, but these changes due to link removal can be overshadowed and lead to a change in $\rho(\boldsymbol{S})$ too small to be captured under finite precision. We observe that this is actually the case in our real-world testbeds: Only 0.005% of contacts are among patients in UVA-PRECOVID dataset, and 0.004% in UVA-COVID dataset.

Minimize the upper bound of $\rho(S)$

Hence we propose an alternate strategy to solve FIP and FCP by minimizing an upper bound on $\rho(\boldsymbol{S})$ that can be computed more precisely. In particular, we define a *system-adjacency* matrix as $\boldsymbol{A} := \prod_{t=1}^{T} \boldsymbol{A}_{T-t+1}$, and minimize its largest eigenvalue $\rho(\boldsymbol{A})$ as a surrogate to minimize the upper bound of $\rho(\boldsymbol{S})$. The following theorem shows that $\rho(\boldsymbol{S})$ is upper bounded by a *monotonic* function of $\rho(\boldsymbol{A})$, justifying our approach of minimizing $\rho(\boldsymbol{A})$ instead.

Theorem 2. *Let $\boldsymbol{S}$, $\boldsymbol{A}$ denote the system and system-adjacency matrices of* 2-MODE-SIS *model with T number of timesteps. Then $\rho(\boldsymbol{S}) \leq f(\rho(\boldsymbol{A}))$, where*

$$f(x) = \frac{1}{2}\Bigg\{(1-\delta)^T + Cx + 1 + \operatorname{sqrt}\Bigg(\left[(1-\delta)^T + Cx + 1\right]^2 - 4\left[(1-\delta)^T(Cx+1) - \alpha\beta(1-\delta)^{2T-2}\right]\Bigg)\Bigg\},$$

$C = \sum_{k=0}^{T-1} \tau_{max}^{T-k}\binom{T}{k}$, and $\tau_{max} = \max_{i \neq j,t}\{\tau_{ijt}\}$. Further, $f(x)$ is a monotonic increasing function in x.

Proof. Lemma 3 first states a general upper bound on the spectral radius of any matrix $\boldsymbol{M}$ structured in a particular way. This Lemma can be proven using Schur complements and the interlacing theorem on a principal submatrix.

Lemma 3. *Given non-negative scalars a, b, c, non-negative $n \times n$ matrix $\boldsymbol{O}$, and $(n+p) \times (n+p)$ matrix $\boldsymbol{M}$:*

$$\boldsymbol{M} = \begin{bmatrix} a\boldsymbol{I}_p & b\boldsymbol{I}_p & \boldsymbol{0} \\ c\boldsymbol{I}_p & \multicolumn{2}{c}{\multirow{2}{*}{$\boldsymbol{O}$}} \\ \boldsymbol{0} & & \end{bmatrix}$$

where $\boldsymbol{I}_p$ denotes the $p \times p$ identity matrix with $p < n$. Then, for the spectral radius of $\boldsymbol{M}$ we have $\rho(\boldsymbol{M}) \leq g(\rho(\boldsymbol{O}), a, b, c)$, where $g(x,a,b,c) = \frac{1}{2}\left(a + x + \sqrt{(a+x)^2 - 4(ax - bc)}\right)$. Further, $g(x,a,b,c)$ is monotonic increasing w.r.t. x.

Proof. Let $\boldsymbol{M}'$ denote a matrix that is almost the same as $\boldsymbol{M}$, but does not have the zeros filling up the gap:

$$\boldsymbol{M}' = \begin{bmatrix} a\boldsymbol{I}_n & b\boldsymbol{I}_n \\ c\boldsymbol{I}_n & \boldsymbol{O} \end{bmatrix}$$

Then, using the Schur complement leads to

$$\begin{aligned} \det(\boldsymbol{M}' - \lambda \boldsymbol{I}) &= \det\left((\boldsymbol{O} - \lambda \boldsymbol{I})(a\boldsymbol{I} - \lambda \boldsymbol{I}) - bc\boldsymbol{I}\right) \\ &= \det\left((a-\lambda)\boldsymbol{O} - (\lambda(a-\lambda) + bc)\boldsymbol{I}\right) \end{aligned}$$

This means that λ is an eigenvalue of $\boldsymbol{M}'$ if and only if $\mu = \dfrac{\lambda(a-\lambda)+bc}{a-\lambda}$ is an eigenvalue of $\boldsymbol{O}$. This is a quadratic equation of λ, of which the solution is

$$\lambda = \frac{1}{2}\left[a + \mu \pm \sqrt{(a+\mu)^2 - 4(a\mu - bc)}\right] =: v(\mu)$$

Next, to show that $\rho(\boldsymbol{M}') = v(\rho(\boldsymbol{A}))$, we show that $v(\cdot)$ is a monotonically non-decreasing function (i.e., $\dfrac{\partial v}{\partial \mu} \geq 0$)

$$\begin{aligned} \frac{\partial v}{\partial \mu} &= 1 \pm \frac{2(a+\mu) - 4a}{2\sqrt{(a+\mu)^2 - 4(a\mu - bc)}} \\ &= 1 \pm \frac{\mu - a}{\sqrt{(a+\mu)^2 - 4(a\mu - bc)}} \\ &= 1 \pm \frac{\mu - a}{\sqrt{(\mu - a)^2 + 4bc}} \\ &\geq 0 \quad (\text{since } b, c \leq 0) \end{aligned}$$

Lastly, note that $\boldsymbol{M}$ is a principle submatrix of $\boldsymbol{M}'$. Since $\boldsymbol{M}'$ is non-negative, applying interlacing theorem of principle submatrix[42] leads to $\rho(\boldsymbol{M}) \leq \rho(\boldsymbol{M}')$ and this proves the lemma. ☐

Using Lemma 3 and the fact that $\boldsymbol{S}$ is structured similarly to $\boldsymbol{M}$, Lemma 4 shows that $\rho(\boldsymbol{S})$ is upper bounded by a monotonic function of $\rho(\boldsymbol{R})$, where $\boldsymbol{R}$ denotes an intermediate *system-transfer* matrix $\boldsymbol{R} := \prod_{t=1}^{T} \boldsymbol{R}_{T-t+1}$.

Lemma 4. *Given $\boldsymbol{S}$, $\boldsymbol{R}$ and $g(\cdot)$ as before, then*

$$\rho(\boldsymbol{S}) \leq g\left(\rho(\boldsymbol{R}), (1-\delta)^T, \alpha(1-\delta)^{T-1}, \beta(1-\delta)^{T-1}\right).$$

Proof. Say we have $T = 2$.

$$\begin{aligned} \boldsymbol{S}_2\boldsymbol{S}_1 &= \begin{bmatrix} (1-\delta)\boldsymbol{I}_p & \beta\boldsymbol{I}_p & \boldsymbol{0} \\ \alpha\boldsymbol{I}_p & \multicolumn{2}{c}{\boldsymbol{R}_2} \\ \boldsymbol{0} & & \end{bmatrix} \begin{bmatrix} (1-\delta)\boldsymbol{I}_p & \beta\boldsymbol{I}_p & \boldsymbol{0} \\ \alpha\boldsymbol{I}_p & \multicolumn{2}{c}{\boldsymbol{R}_1} \\ \boldsymbol{0} & & \end{bmatrix} \\ &= \begin{bmatrix} ((1-\delta)^2 + \alpha\beta)\boldsymbol{I}_p & \begin{bmatrix}\beta\boldsymbol{I}_p & \boldsymbol{0}\end{bmatrix} + \beta\boldsymbol{R}_1^{prow} \\ \begin{bmatrix}\alpha\boldsymbol{I}_p \\ \boldsymbol{0}\end{bmatrix} + \alpha\boldsymbol{R}_2^{pcol} & \boldsymbol{R}_2\boldsymbol{R}_1 + \begin{bmatrix}\alpha\beta\boldsymbol{I}_p & \boldsymbol{0} \\ \boldsymbol{0} & \boldsymbol{0}\end{bmatrix} \end{bmatrix} \\ &\approx \begin{bmatrix} (1-\delta)^2\boldsymbol{I}_p & \beta(1-\delta)\boldsymbol{I}_p & \boldsymbol{0} \\ \alpha(1-\delta)\boldsymbol{I}_p & \multicolumn{2}{c}{\boldsymbol{R}_2\boldsymbol{R}_1} \\ \boldsymbol{0} & & \end{bmatrix} \end{aligned}$$

For $T > 2$, repeatedly multiplying and omitting high-order terms leads to following approximation:

$$\boldsymbol{S} \approx \begin{bmatrix} (1-\delta)^T \boldsymbol{I}_p & \beta(1-\delta)^{T-1}\boldsymbol{I}_p & \boldsymbol{0} \\ \alpha(1-\delta)^{T-1}\boldsymbol{I}_p & \multicolumn{2}{c}{\multirow{2}{*}{\boldsymbol{R}}} \\ \boldsymbol{0} & & \end{bmatrix}$$

This approximation has the exact structure discussed in Lemma 3. Applying the Lemma proves the claim. □

Note that having less non-infectable nodes leads to a tighter upper bound. Hence, starting from Lemma 4, we can obtain an upper bound on $\rho(\boldsymbol{S})$ in terms of $\rho(\boldsymbol{A})$ as such:

$$\begin{aligned} \rho(\boldsymbol{S}) &\leq g(\rho(\boldsymbol{R}), (1-\delta)^T, \alpha(1-\delta)^T, \beta(1-\delta)^T) \\ &\leq g(C\rho(\boldsymbol{A})+1, (1-\delta)^T, \alpha(1-\delta)^T, \beta(1-\delta)^T) \end{aligned}$$

The second inequality is due to monotonicity of $g(\cdot)$ with respect to the first input, and $\rho(\boldsymbol{R}) \leq C\rho(\boldsymbol{A}) + 1$ from Lemma 5. Expanding the last term leads to the desired result.

Next, Lemma 5 shows that $\rho(\boldsymbol{R})$ is also upper bounded by a monotonic function of $\rho(\boldsymbol{A})$ (which contains only zeros and ones) by viewing $\boldsymbol{R}$ as a Hadamard product of a constant matrix times $\boldsymbol{A}$, plus some diagonal terms.

Lemma 5. *Given $\boldsymbol{R}$, $\boldsymbol{A}$ as before, $\rho(\boldsymbol{R})$ is upper bounded by a monotonic function of $\rho(\boldsymbol{A})$: $\rho(\boldsymbol{R}) \leq C\rho(\boldsymbol{A}) + 1$ (C and τ_{max} as given in Theorem 2).* □

Proof. Note that the individual transfer matrices $\boldsymbol{R}_t$ are formed by a Hadamard product plus diagonal terms: $\boldsymbol{R}_t = \boldsymbol{T}_t \circ \boldsymbol{A}_t + \boldsymbol{D}_t$. Here $\boldsymbol{T}_t$ stores the transfer rates $[\boldsymbol{T}_t]_{i,j} = \tau_{ijt}$, and $\boldsymbol{D}_t$ are the additional diagonal terms. Then, the product $\boldsymbol{R}$ can be written as

$$\begin{aligned} \boldsymbol{R} = \prod_{t=1}^{T} \boldsymbol{R}_t = \prod_{t=1}^{T} \boldsymbol{T}_t \circ \boldsymbol{A}_t + \boldsymbol{D}_t &\leq \prod_{t=1}^{T} \tau_{max}\boldsymbol{A}_t + \boldsymbol{I} \\ &= \tau_{max}^T \prod_{t=1}^{T} \boldsymbol{A}_t + \tau_{max}^{T-1} \sum_{\substack{S \subset \{1,\ldots,T\} \\ |S| = T-1}} \prod_{t \in S} \boldsymbol{A}_t + \cdots + \boldsymbol{I} \\ &\leq \tau_{max}^T \boldsymbol{A} + \tau_{max}^{T-1} \binom{T}{1} \boldsymbol{A} + \cdots + \boldsymbol{I} \\ &= \sum_{k=0}^{T-1} \tau_{max}^{T-k} \binom{T}{k} \boldsymbol{A} + \boldsymbol{I} \end{aligned}$$

where the inequalities are element-wise. Then using Weyl's inequality leads to the desired upper-bound on $\rho(\boldsymbol{R})$. □

Theorem 2 then follows from Lemma 4 and Lemma 5 due to monotonicity of the upper bounds. □

We observe that the upper bound follows our intuition with respect to individual parameters: A large α,β or small δ leads to a large upper bound, implying possibility of a large $\rho(\boldsymbol{S})$. Increasing $\rho(\boldsymbol{A})$ also increases the upper bound, but how much it increases depends on τ_{max} or how much pathogen can be transferred through a single contact. Leveraging Theorem 2, we propose to solve the following two surrogate problems:

Surrogate Isolation Problem (SIP). *Let $\boldsymbol{A}(P)$ denote the system-adjacency matrix with patients in set P under precaution. Given all patients X and the budget constraint k, find a subset of patients P^* for precaution s.t.*

$$P^* = \arg\min_P \rho(\boldsymbol{A}(P)) \text{ subject to } P \subset X \text{ and } |P| = k.$$

Surrogate Clearance Problem (SCP). *Let $\boldsymbol{A}(\cdot)$ be as in SIP. Given an initial set Q of d candidate patients already under precaution ($|Q| = d$) and a budget constraint $k < d$, find the optimal subset of patients $P^* \subset Q$ for clearance:*

$$P^* = \arg\min_P \rho(\boldsymbol{A}(Q \setminus P)) \text{ subject to } P \subset Q \text{ and } |P| = k.$$

Final algorithm

Fortunately, $\rho(\boldsymbol{A})$ does not suffer from the same numerical issue as $\rho(\boldsymbol{S})$ does. However, the two surrogate problems are also NP-hard. Hence we propose a simple yet effective heuristic algorithm, GREEDY-SPECTRAL, for SIP and SCP: Starting from an empty set, we iteratively find and add a patient for isolation (or releasing) that leads to the smallest $\rho(\boldsymbol{A})$ value until the size of the set reaches the given budget. Note that in each iteration of GREEDY-SPECTRAL, we need to recalculate the change in $\rho(\boldsymbol{A})$ after isolating or releasing every node, requiring $O(kN)$ number of $\rho(\boldsymbol{A})$-computations. Each $\rho(\boldsymbol{A})$-computation can be time-consuming when done naively through computing and storing the full dense $\boldsymbol{A}$ and then computing its spectral radius, which takes $O(N^{2.37}T)$ time[43]. Instead, we leverage the sparsity of individual $\boldsymbol{A}_t$, and use power iterations to compute $\rho(\boldsymbol{A})$ without explicitly constructing $\boldsymbol{A}$. The following Lemma 6 states the computational cost of this approach.

Lemma 6. *Computing $\rho(\boldsymbol{A})$ with the Arnoldi method using implicit linear operator $\boldsymbol{A}_{op}$ requires $O(\sum_{t=1}^{T} \text{nnz}(\boldsymbol{A}_t))$ time and $O(N)$ space per iteration, where $\text{nnz}(\boldsymbol{A}_t)$ denotes the number of nonzeros in $\boldsymbol{A}_t$.*

Proof. Since the Arnoldi method only requires a function that computes matrix-vector products with the query matrix, we can use a linear operator $\boldsymbol{A}_{op} : \boldsymbol{x} \mapsto \boldsymbol{A}_T \cdots \boldsymbol{A}_1 \boldsymbol{x}$ that does not explicitly compute $\boldsymbol{A}$ and then apply to $\boldsymbol{x}$, but rather applies $\boldsymbol{A}_1, \boldsymbol{A}_2$, up to $\boldsymbol{A}_T$ one-by-one to the input vector $\boldsymbol{x}$.

Hence, when searching for the largest-magnitude eigenvalue of $\boldsymbol{A}$, each i-th iteration of the Arnoldi method first computes the matrix-vector product $\boldsymbol{q} = \boldsymbol{A}_{op}(\boldsymbol{v}_i)$ and then normalizes the result $\boldsymbol{v}_{i+1} = \frac{\boldsymbol{q}}{\|\boldsymbol{q}\|}$. Thus, each iteration only needs to store a length-N vector, which leads to the $O(N)$ space complexity. The time complexity is dominated by the cost of computing $\boldsymbol{A}_{op}(\boldsymbol{v})$. Within $\boldsymbol{A}_{op}$, computing the sparse matrix-vector product $\boldsymbol{A}_t\boldsymbol{v}$ requires $O(\text{nnz}(\boldsymbol{A}_t))$ time: each term in $\boldsymbol{A}_t$ is multiplied by an entry in $\boldsymbol{v}$ and added to the output vector only once. Therefore, aggregating the cost of T sparse matrix-vector products in $\boldsymbol{A}_{op}$ leads to $O(\sum_{t=1}^{T} \text{nnz}(\boldsymbol{A}_t))$ time. □

When using the Arnoldi method, the overall computational cost of GREEDY SPECTRAL can be reduced down to $O(kn\sum_{t=1}^{T} \text{nnz}(\boldsymbol{A}_t))$-time and $O(N)$-space complexity as shown in Supplementary Fig 3. Additionally, the change in $\rho(\boldsymbol{A})$ after adding each node to the solution set can be computed in parallel for further speedup.

Experiemnts setup

Datasets: We evaluate the performance of GREEDY-SPECTRAL on various real datasets (see Supplementary Table 3, the meaning of notations are listed in Supplementary Table 1). On each dataset, we run 1000 simulation runs and report the average. For UVA datasets, we run the simulation till 294 timesteps (covering the entire given time period). This dataset contains a large suite of clinical metadata from the Epic-based SQL database at the University of Virginia (UVA) hospital. It also contains the weekly number of new tested positive MRSA cases in the hospital, and a rich set of features for each patient. From the dataset, we construct two time-varying heterogeneous contact networks, one representing 294 days before COVID-19 (May 2019 to Feb 2020), referred as the UVA-PRECOVID network, and another representing 294 days since the pandemic (May 2020 to Feb 2021), referred as the UVA-COVID network. The networks are derived from inpatient Data, doctor's Notes, and MedAdmin Data, which record the time and location of interactions between patients and HCWs. We aggregate these into daily networks such that two nodes are connected in $\mathscr{G}_t$ if they have at least one contact at day t.

Besides, we also use a smaller dataset, LYON, to showcase the tipping point as in Supplementary Fig 1. This dataset is collected using wearable RFID sensors[44,45] and contacts were recorded when people are in close proximity. Similarly to UVA, we construct $\mathscr{G}_t$ if they have at least one contact at day t. Specifically, we use this dataset to show that our GREEDY-SPECTRAL algorithm for minimizing $\rho(\boldsymbol{A})$ is also effective in minimizing $\rho(\boldsymbol{S})$. However, this dataset doesn't contain the number of new tested positive MRSA cases, so we will use synthetic parameters in the experiments.

Baselines: We compare GREEDY-SPECTRAL algorithm against the following popular methods:

- RANDOM: Randomly pick k patients.
- PROPDEGREE: Pick k patients with the probability of picking i proportional to the sum of the nonzero terms of i in R_t (intuitively, the degree of i) over all timesteps (i.e., $p \propto \sum_t \sum_j \mathbb{1}(R_t(j,i))$).
- SHORR: Pick k patients with the highest so-called Shorr scores[46]; Shorr score was developed to assess the clinical risk of an admitted patient acquiring MRSA, and variables used include the history of recent hospitalizations or kidney dialysis. This requires access to EHRs and is not applicable to the LYON dataset. Note that Shorr score is one of the classical clinical criteria used to pick patients for MRSA contact precaution[46], we compare with SHORR to showcase that our GREEDY-SPECTRAL is more effective than such usual practices using patient-level features.
- OPTIMALSPECTRAL: Pick the optimal k patients that minimize $\rho(\boldsymbol{A})$ by testing all possible combinations. However, this method is not scalable on large networks and we only get the results on the LYON testbed.

Evaluation metrics: We use the following metrics to quantify the performance in suppressing MRSA outbreak.

- $\eta_{\rho(\boldsymbol{A})}$: Ratio of $\rho(\boldsymbol{A})$ with $\mathscr{P}$ under precaution against $\rho(\boldsymbol{A})$ when $k=0$ (no patient under precaution) (i.e. $\eta_{\rho(\boldsymbol{A})} = \frac{\rho(\boldsymbol{A}(\mathscr{P}))}{\rho(\boldsymbol{A}(\emptyset))}$). Lower is better.
- γ_{CASES}: Ratio of the number of new MRSA cases against $k=0$. (e.g. γ_{CASES} for PROPDEGREE is $\frac{\text{CASES}_{\text{PROPDEGREE}}}{\text{CASES}_{k=0}}$, where $\text{CASES}_{\text{PROPDEGREE}}$ and $\text{CASES}_{k=0}$ are the number of new MRSA cases of PROPDEGREE and $k=0$ respectively). Lower γ_{CASES} is better.
- γ_{LOADS}: Ratio of non-patient (HCWs and location) pathogen loads against $k=0$. (e.g. γ_{LOADS} for PROPDEGREE is $\frac{\text{LOADS}_{\text{PROPDEGREE}}}{\text{LOADS}_{k=0}}$, where $\text{LOADS}_{\text{PROPDEGREE}}$ and $\text{LOADS}_{k=0}$ are the sum of HCWs pathogen loads and location pathogen loads of PROPDEGREE and $k=0$ respectively). Lower γ_{CASES} is better.
- PROB[CASES $>$ TARGET]: Probability that the number of new MRSA cases is larger than TARGET. (e.g. PROB[CASES $>$ 500] is the probability that the number of new MRSA cases is larger than 500). Lower PROB[CASES $>$ TARGET] is better.

Additional results

First, we show how GREEDY-SPECTRAL performs in minimizing $\rho(\boldsymbol{A})$ for Isolation problem. As seen in Supplementary Fig 4, our algorithm achieves lower $\eta_{\rho(\boldsymbol{A})}$ than RANDOM, PROPDEGREE, and SHORR on both UVA-PRECOVID and UVA-COVID dataset, as isolation budget k increases. This demonstrates the effectiveness of our algorithm in minimizing $\rho(\boldsymbol{A})$.

Next, we show how GREEDY-SPECTRAL performs in minimizing $\rho(\boldsymbol{A})$ for Clearance problem. As seen in Supplementary Fig 5, our algorithm achieves lower $\eta_{\rho(\boldsymbol{A})}$ than RANDOM, PROPDEGREE, and SHORR on both UVA-PRECOVID and UVA-COVID dataset, as clearance budget k increases. This demonstrates the effectiveness of our algorithm in minimizing $\rho(\boldsymbol{A})$.

High precaution and cleaning scenario

Additional isolation problem results: Here, we first show more experiment results for high precaution and cleaning scenario (main article Fig 3) in Supplementary Fig 6. In the main article Fig 3, we only show the results of γ_{LOADS} and γ_{CASES} for $k = 1000, 2000, 3000, 4000, 5000$ using bars. In Supplementary Fig 6, we also add $k = 500, 1500, 2500, 3500, 4500$ results and show them using lines.

Clearance problem results: Here, we show the experiments for the clearance problem (CP) under high precaution and and cleaning scenario. Supplementary Fig 7 shows how GREEDY-SPECTRAL performs in minimizing non-patient pathogen loads and the number of new MRSA cases. As shown in Supplementary Fig 7(a), the ratio of non-patient pathogen loads γ_{LOADS} is always lower for our algorithm than other baselines. For example, when $k = 4000$, it achieves 9.2% (10.8%) lower loads than SHORR on UVA-PRECOVID (UVA-COVID). This indicates that our algorithm is more effective in reducing the non-patient pathogen loads than other baselines. However, in Supplementary Fig 7(b), we observe that it still gives a similar ratio of new MRSA cases γ_{CASES} as other baselines. Similarly, in Supplementary Fig 7(c), it also gives similar PROB[CASES $>$ TARGET] for varying TARGET (x-axis) as other baselines.

Relaxed precaution and cleaning scenario

Isolation problem results:

First, we show more experiment results for relaxed precaution and cleaning scenario with fixing α such that the number of cases is 1000 when $k=0$ but changing the k (main article Fig 4) in Supplementary Fig 8.

Next, we will show more results by fixing k but changing α. Supplementary Fig 9 shows the results with varying α but fixing k. As shown in Supplementary Fig 9, GREEDY-SPECTRAL achieves lower non-patient pathogen loads and number of new MRSA cases than other baselines. For example, for α such that the number of cases is 900 when $k=0$ in UVA-COVID (bottom row), it leads to 12.6% less loads in Supplementary Fig 9(a) and 9.2% less cases in Supplementary Fig 9(b). In Supplementary Fig 9(c), our algorithm can always lead to less than 500 cases, while SHORR has more than 48.6% probability of having more than 500 cases. From Supplementary Fig 9, we can conclude GREEDY-SPECTRAL algorithm is effective in suppressing the MRSA outbreak under the relaxed precaution and cleaning scenario for isolation problem.

Clearance problem results:

Similarly, we first show more experiment results for relaxed precaution and cleaning scenario with fixing α such that the number of cases is 1000 when $k=0$ but changing the k (main article Fig 5) in Supplementary Fig 10.

Then we show more results by fixing k but changing α. Supplementary Fig 11 shows the results with varying α but fixing k. As shown in Supplementary Fig 11, GREEDY-SPECTRAL achieves lower non-patient pathogen loads and number of new MRSA cases than other baselines. For example, for α such that the number of cases is 700 when $k=0$ in UVA-PRECOVID (top row), it leads to 16.8% less loads than other baselines in Supplementary Fig 11(a) and 11.2% less cases in Supplementary Fig 11(b). In Supplementary Fig 11(c), our algorithm has only 5.0% probability of having more than 500 cases, while other

baselines have more than 90.5% probability of having more than 500 cases. FromSupplementary Fig 11 into consideration, we can conclude GREEDY-SPECTRAL algorithm is effective in suppressing the MRSA outbreak under the relaxed precaution and cleaning scenario for clearance problem.

Performance of GREEDY-SPECTRAL in minimizing $\rho(\boldsymbol{S})$

Supplementary Fig 12 shows the effectiveness of minimizing $\rho(\boldsymbol{S})$ by minimizing $\rho(\boldsymbol{A})$ using GREEDY-SPECTRAL. In Supplementary Fig 12, we compare performance between the result of our algorithm in minimizing $\rho(\boldsymbol{A})$ and OPTIMALSPECTRAL method in minimizing $\rho(\boldsymbol{S})$ directly. Here, we perform the experiments on LYON dataset with synthetic parameters since the LYON dataset is the only dataset scalable for OPTIMALSPECTRAL while having no ground-truth number of cases for calibration. As shown in Supplementary Fig 12, we can see the two methods achieve similar performance in $\eta_{\rho(\boldsymbol{S})}$ (note that the two lines are overlapping), which demonstrates the effectiveness of our GREEDY-SPECTRAL algorithm in minimizing $\rho(\boldsymbol{S})$.

SIP and FIP are NP-hard

Proof. Note that the Nodal Spectral Radius Minimization (NSRM) problem[41] is a special case of SIP where all nodes are infectable and there is only one timestep (i.e., $|V| = P$ and $T = 1$). This implies SIP is at least as hard as NSRM, and is thus NP-hard.

Next, we prove FIP is NP-hard by showing FIP is a generalization of SIP proven to be NP-hard above. For all $t = 1,\dots,T$, each system matrix $\boldsymbol{S}_t$ equals adjacency $\boldsymbol{A}_t$ with zero-padding when $(\alpha,\beta,\delta) = (0,0,1)$, $\tau_{ijt} = 1$ for all $i \neq j$, and $\tau_{ijt} = 0$ for all $i = j$. Given this setup, $\boldsymbol{S}$ encodes $\boldsymbol{A}$ within the bottom-right block. Below is an illustrative example with $T = 2$:

$$\boldsymbol{S} = \boldsymbol{S}_1\boldsymbol{S}_2 = \begin{bmatrix} 0 & 0 & 0 \\ 0 & \multicolumn{2}{c}{\multirow{2}{*}{$\boldsymbol{A}_1$}} \\ 0 & & \end{bmatrix} \begin{bmatrix} 0 & 0 & 0 \\ 0 & \multicolumn{2}{c}{\multirow{2}{*}{$\boldsymbol{A}_2$}} \\ 0 & & \end{bmatrix} = \begin{bmatrix} 0 & 0 & 0 \\ 0 & \multicolumn{2}{c}{\multirow{2}{*}{$\boldsymbol{A}_1\boldsymbol{A}_2$}} \\ 0 & & \end{bmatrix} = \begin{bmatrix} 0 & 0 & 0 \\ 0 & \multicolumn{2}{c}{\multirow{2}{*}{$\boldsymbol{A}$}} \\ 0 & & \end{bmatrix}$$

Note that this block-structure enforces not only $\rho(\boldsymbol{S}) = \rho(\boldsymbol{A})$, but also $\rho(\boldsymbol{S}(\mathscr{P})) = \rho(\boldsymbol{A}(\mathscr{P}))$ for any possible set of isolated patients $\mathscr{P}$. This implies existence of an FIP instance that is equivalent to any given instance of SIP. Hence FIP is at least as hard as SIP, and is thus NP-hard. □

SCP and FCP are NP-hard

Proof. We prove this by reducing the Independent Set (IS) problem to SCP. Given a graph $G = (V,E)$, the goal of IS is to find a set of $k \leq |V|$ nodes that do not share an edge. Note that a graph with no links between nodes has the all-zero adjacency matrix and a spectral radius of 0. Thus given an instance of IS, we can construct an equivalent instance of SCP by using the same graph G with all nodes representing infectable patients initially under precaution (i.e., $\mathscr{R} = V$), and then aiming to clear out $|V| - k$ patients from precaution. If the resulting graph with k patients still under precaution has spectral radius equal to 0, then the graph G contains an independent set of k nodes. If the smallest achievable spectral radius is larger than 0, then no independent set of size k exists. This implies SCP is at least as hard as IS, and is thus NP-hard.

Then leveraging the exact same setting as for SIP and FIP above, we can reduce an instance of SCP to an equivalent instance of FCP. Therefore, FCP is at least as hard as SCP, and hence FCP is NP-hard. □

Parameter setup

As mentioned in the main article, for high precaution and cleaning scenario, we calibrate on the weekly number of new tested positive MRSA cases in UVA hospital to get the parameters for 2-MODE-SIS model. For calibration, we use the Approximate Bayesian Computation-Rejection scheme implemented in the ABCPY package[47]. The procedure first samples a parameter set Θ_i from a user-defined prior, runs multiple simulations with Θ_i, and then stores Θ_i if the average simulation results are close to actual observations. The final list of Θ_i's then approximates the posterior distribution given actual data. When evaluating which Θ_i to store, we use cumulative weekly counts of new MRSA infections and measure the RMSE error between simulated and actual values. Note that in actual data, only patients who were negative in a previous test but were positive in a later test count as *new* MRSA infections.

Here, we show the effectiveness of the calibration procedure from two perspectives: On the one hand, we use some synthetic parameters to simulate some synthetic MRSA case curves. We then calibrate on these synthetic curves to show that our procedure could "recover" synthetic parameters via calibration. On the other hand, we also calibrate on the real-world MRSA case curves, and show the simulated curves using our calibrated parameters align closely with the real-world curves.

In Supplementary Fig 13, we set up 5 different "scenarios" for synthetic experiments. In each scenario, we set some “ground truth” parameters and run simulations with these ground-truth parameters to generate synthetic MRSA cases curve. Next, we

use these curves for calibration to get the calibrated parameters. If the calibrated parameters closely align with the ground truth parameters we initially set (i.e., we can "recover" these parameters through calibration), we can conclude that the calibration process is effective.

In Supplementary Fig 14, we show that the resulting calibrated parameters fit the model closely towards actual observations. We also list the value of calibrated parameters in Supplementary Table 4. Specifically, in this table, τ_{p2p} refers to the τ_{ijt} for any t where i and j are patients. Others are similarly defined. p, h, l represent patients, HCWs, and locations, respectively.

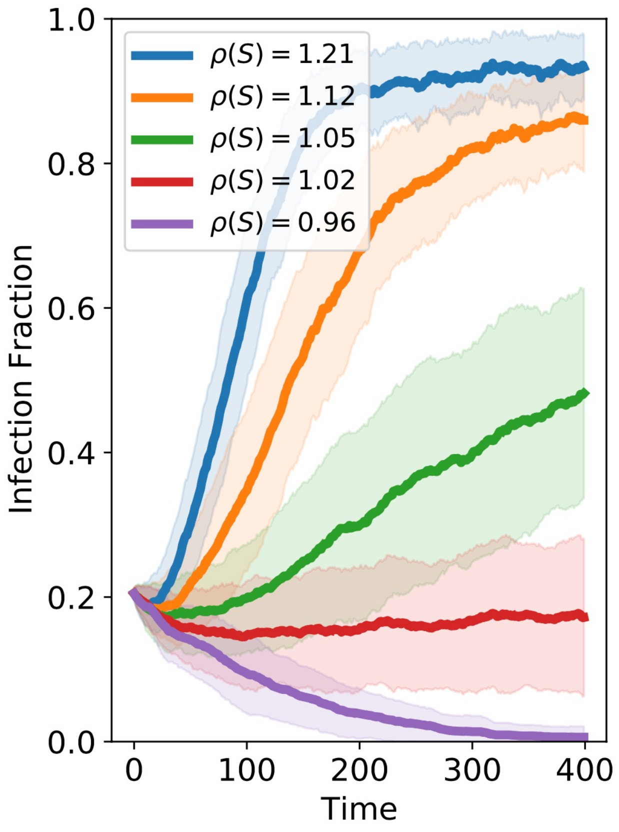

(a) Time vs. Infected fraction

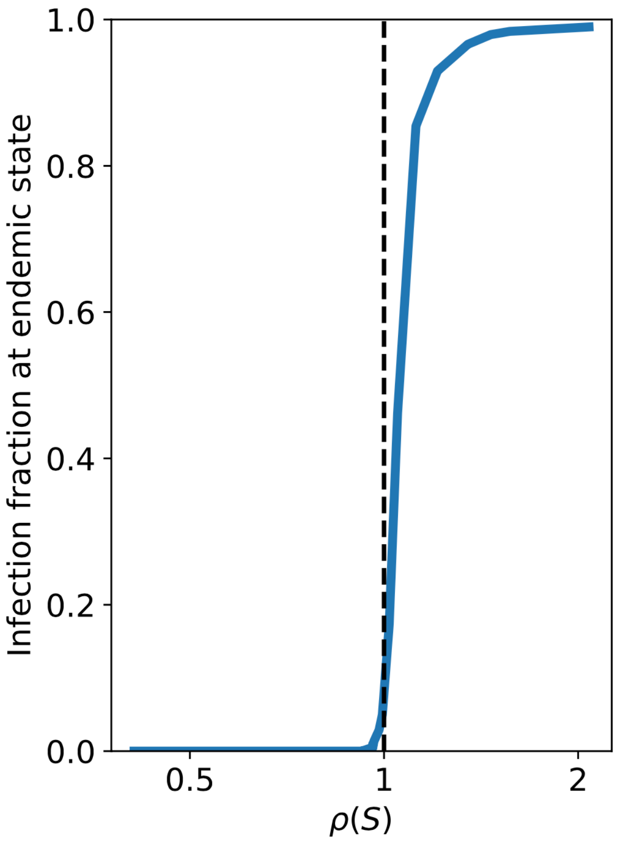

(b) $\rho(\boldsymbol{S})$ vs. Footprint

Supplementary Fig. 1. 2-MODE-SIS simulations on the LYON testbed. (a) Time vs. infected fraction. Banded regions indicate one standard error from an average of 100 runs. The endemic infection size decreases as we reduce $\rho(\boldsymbol{S})$ from 1.21 to 0.96, where the disease goes extinct. (b) $\rho(\boldsymbol{S})$ vs. endemic state infection fraction in log-linear scale. The long-term dynamics shifts at $\rho(\boldsymbol{S}) = 1$ (dotted line).

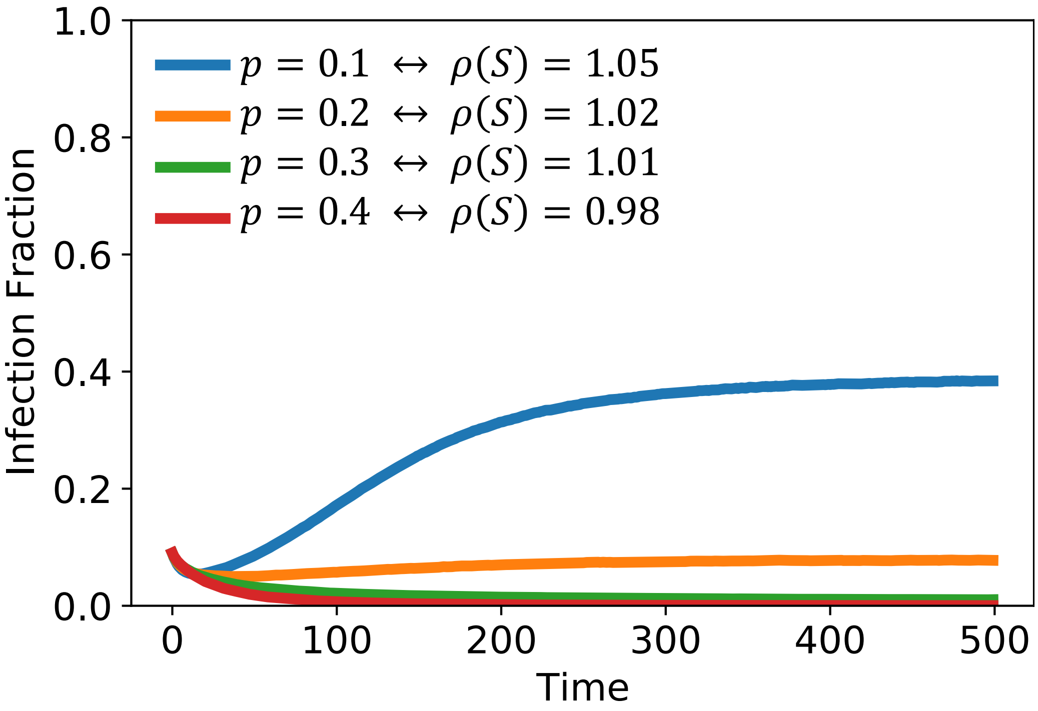

Supplementary Fig. 2. Example of 'dilution effect' on static Erdős-Rényi random graphs. With increasing link probability p, the simulation results show that the infection fraction decreases.

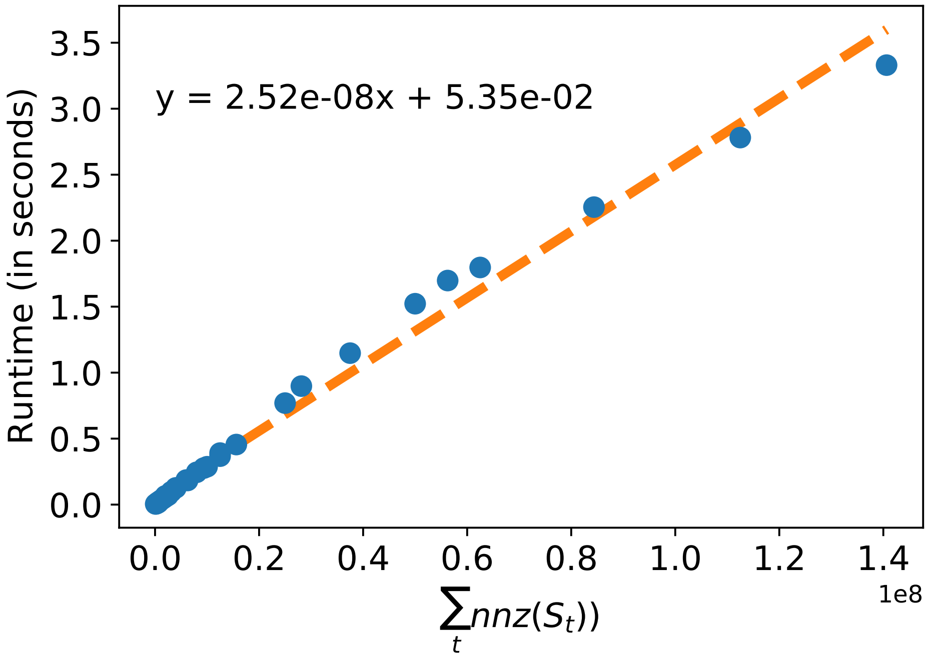

Supplementary Fig. 3. Runtime plot of computing $\rho(\boldsymbol{A})$ with the Arnoldi method. The actual runtimes (in blue) increase linearly to the total number of nonzeros in the $\mathbf{A}_t$ matrices.

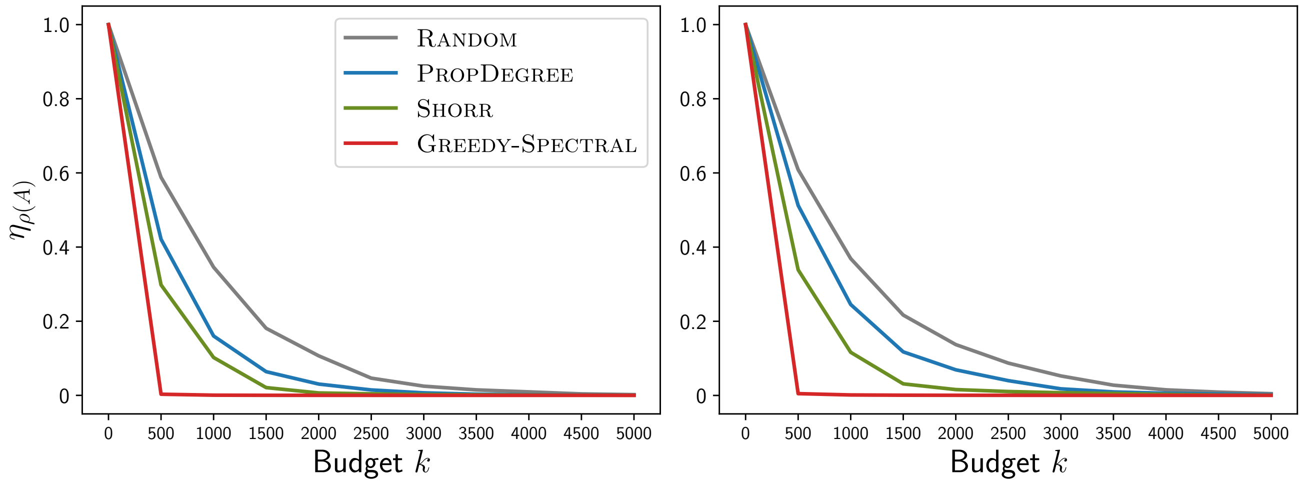

(a) UVA-Precovid (b) UVA-Covid

Supplementary Fig. 4. Change in $\eta_{\rho(\boldsymbol{A})}$ (y-axis) with increasing isolation budget k (x-axis). (a) is for UVA-PRECOVID and (b) is for UVA-COVID. Lower is better.

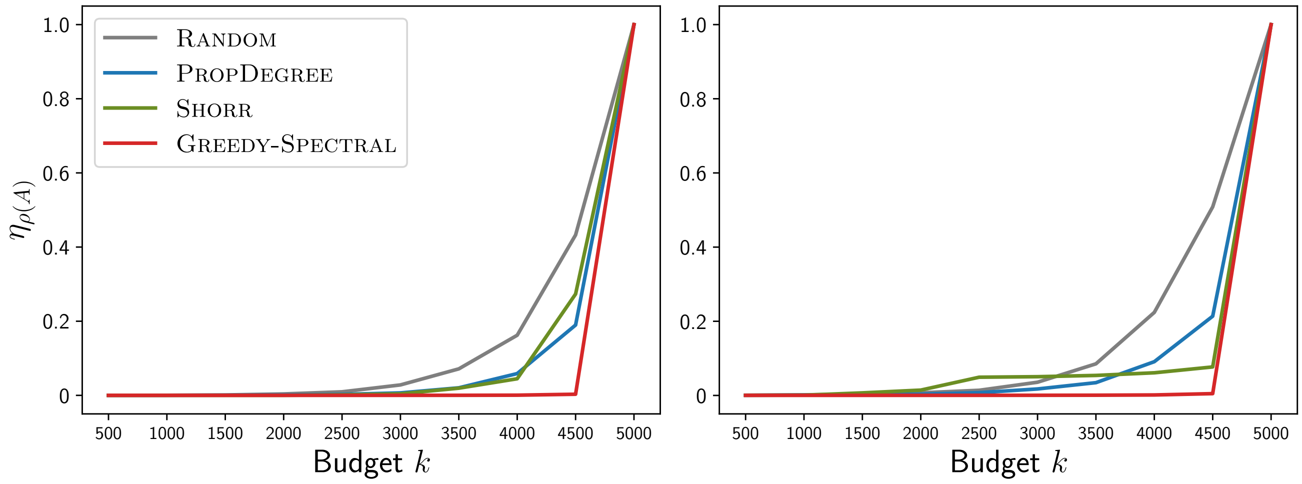

(a) UVA-Precovid (b) UVA-Covid

Supplementary Fig. 5. Change in $\eta_{\rho(\boldsymbol{A})}$ (y-axis) with increasing clearance budget k (x-axis). (a) is for UVA-Precovid and (b) is for UVA-Covid. Lower is better.

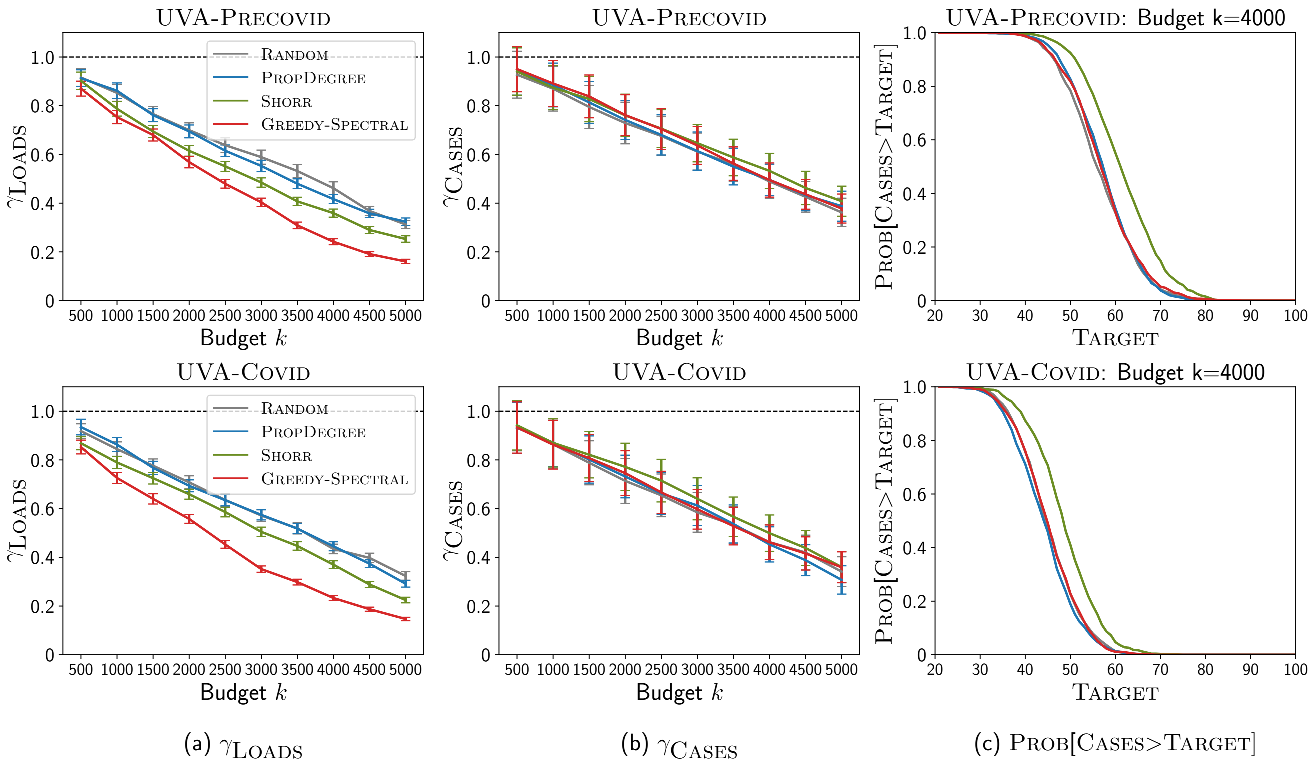

Supplementary Fig. 6. GREEDY-SPECTRAL achieves lower non-patient pathogen loads but similar number of new MRSA cases than other baselines under high precaution and cleaning scenario for isolation problem (IP). Top is for UVA-PRECOVID dataset, down is for UVA-COVID dataset. (a) Ratio of non-patient pathogen loads γ_{LOADS} (y-axis) with varying budget k (x-axis). Lower is better. The bars show the standard error. (b) Ratio of new MRSA cases γ_{CASES} (y-axis) with varying budget k (x-axis). Lower is better. (c) PROB[CASES > TARGET] (y-axis) with varying TARGET (x-axis) for budget $k = 4000$. Lower is better.

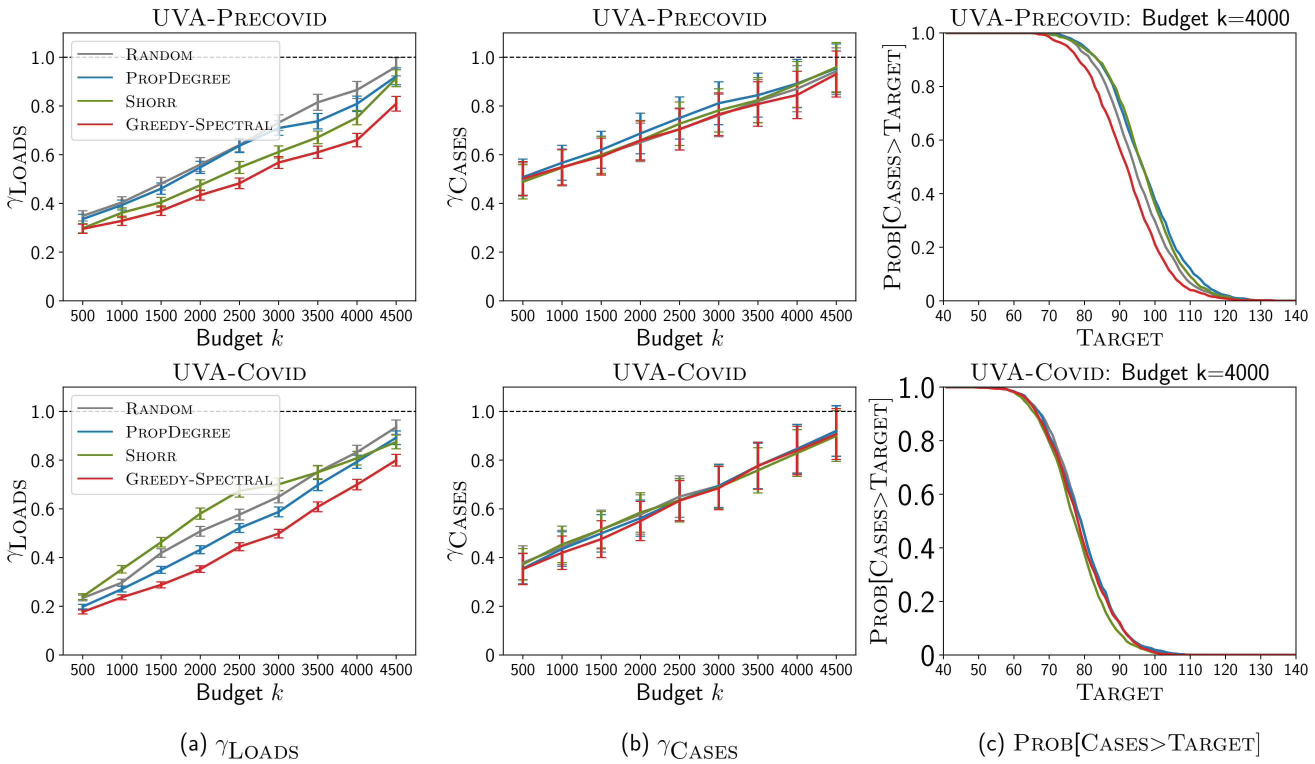

Supplementary Fig. 7. GREEDY-SPECTRAL achieves lower non-patient pathogen loads but similar number of new MRSA cases than other baselines under high precaution and cleaning scenario for clearance problem (CP). Top is for UVA-PRECOVID dataset, down is for UVA-COVID dataset. (a) Ratio of non-patient pathogen loads γ_{LOADS} (y-axis) with varying budget k (x-axis). Lower is better. The bars show the standard error. (b) Ratio of new MRSA cases γ_{CASES} (y-axis) with varying budget k (x-axis). Lower is better. (c) PROB[CASES > TARGET] (y-axis) with varying TARGET (x-axis) for budget $k = 4000$. Lower is better.

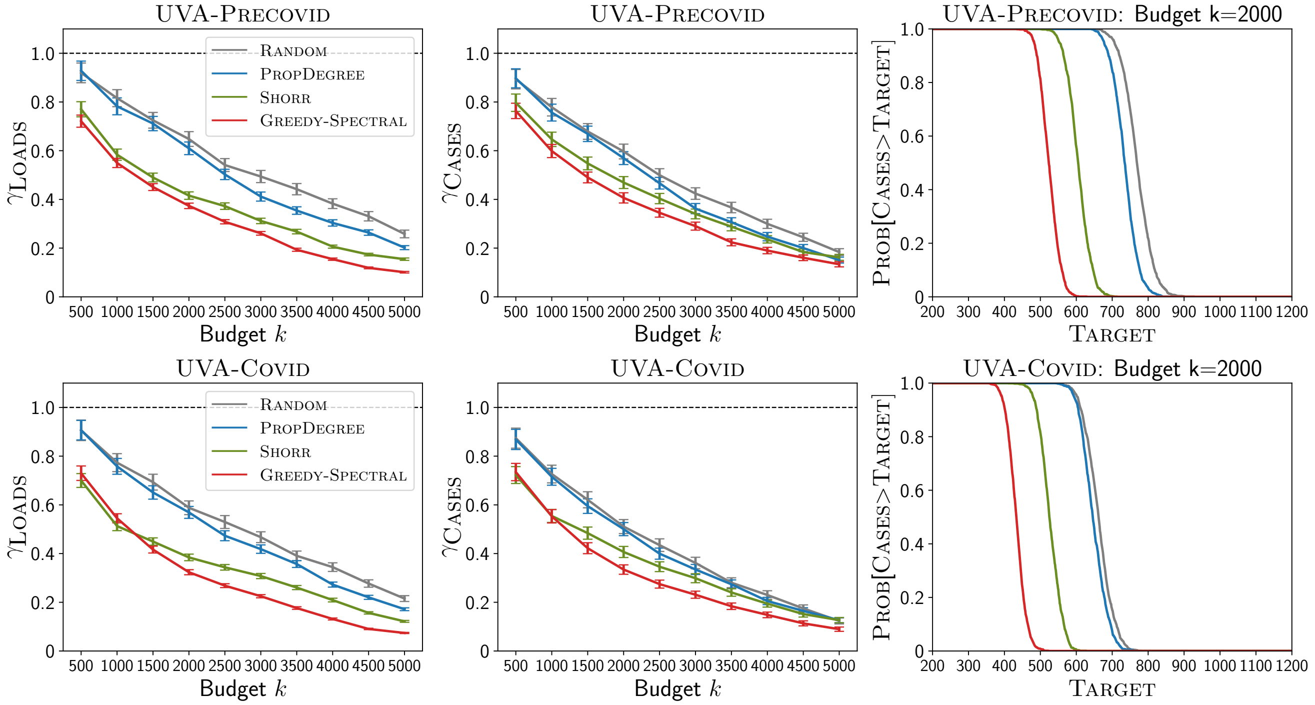

(a) γ_{LOADS} (b) γ_{CASES} (c) PROB[CASES>TARGET]

Supplementary Fig. 8. GREEDY-SPECTRAL achieves lower non-patient pathogen loads and number of new MRSA cases than other baselines under relaxed precaution and cleaning scenario for isolation problem (IP). Top is for UVA-PRECOVID dataset, down is for UVA-COVID dataset. (a) Ratio of non-patient pathogen loads γ_{LOADS} (y-axis) with varying budget k (x-axis). (b) Ratio of new MRSA cases γ_{CASES} (y-axis) with varying budget k (x-axis). (c) PROB[CASES $>$ TARGET] (y-axis) with varying TARGET (x-axis) for budget $k = 2000$.

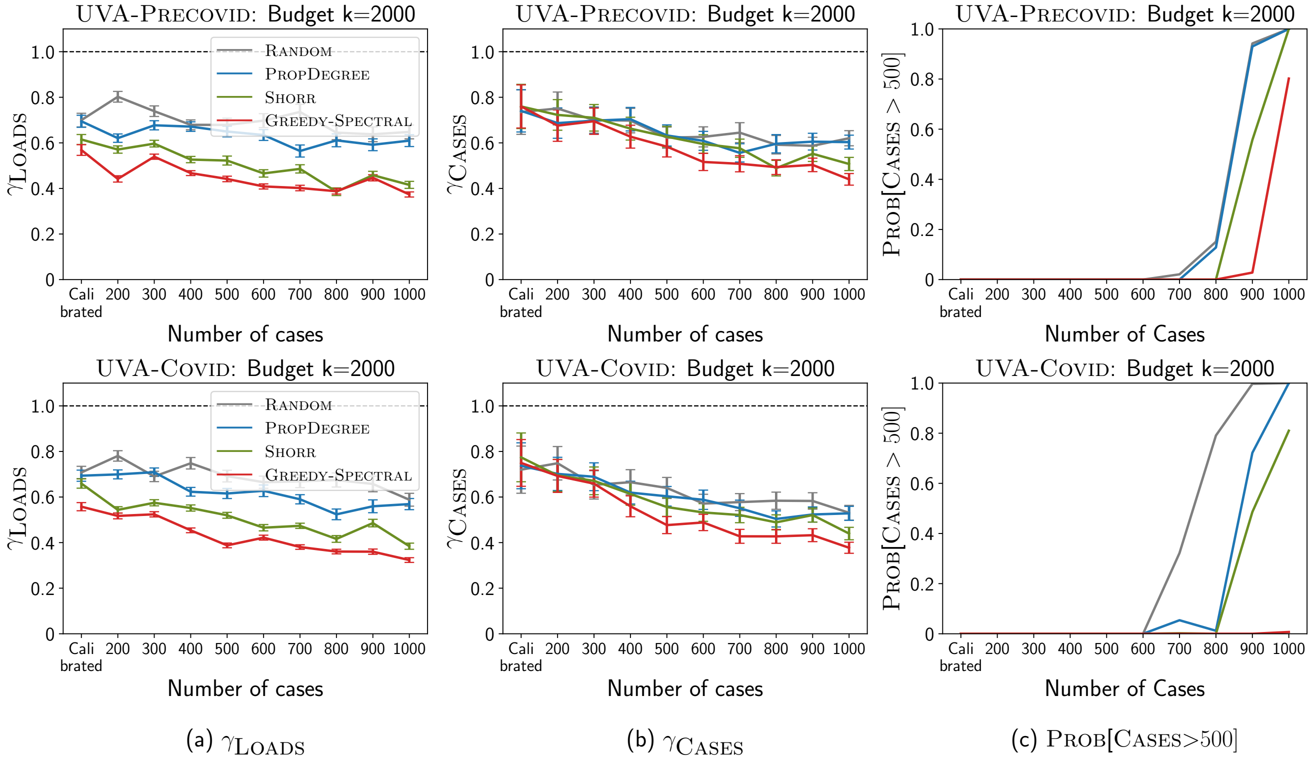

Supplementary Fig. 9. Greedy-Spectral achieves lower non-patient pathogen loads and number of new MRSA cases than other baselines with varying α under relaxed precaution and cleaning scenario for isolation problem (IP). Top is for UVA-Precovid dataset, down is for UVA-Covid dataset. Here we fix the $k = 2000$. (a) Ratio of non-patient pathogen loads γ_{Loads} (y-axis) with varying α (x-axis). (b) Ratio of new MRSA cases γ_{Cases} (y-axis) with varying α (x-axis). (c) Prob[Cases > 500] (y-axis) with varying α (x-axis) for budget $k = 2000$.

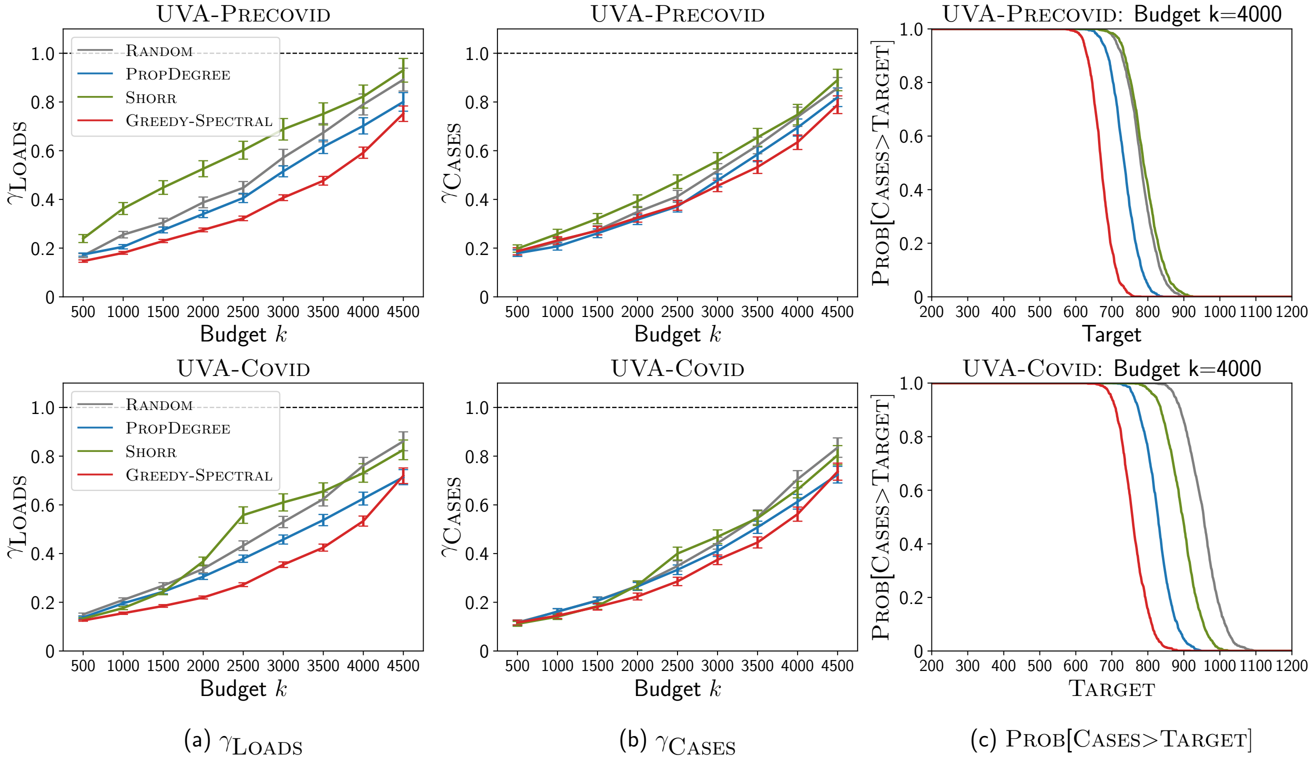

Supplementary Fig. 10. Greedy-Spectral achieves lower non-patient pathogen loads and number of new MRSA cases than other baselines under relaxed precaution and cleaning scenario for clearance problem (CP). Top is for UVA-Precovid dataset, down is for UVA-Covid dataset. (a) Ratio of non-patient pathogen loads γ_{Loads} (y-axis) with varying releasing budget k (x-axis). (b) Ratio of new MRSA cases γ_{Cases} (y-axis) with varying releasing budget k (x-axis). (c) Prob[Cases $>$ Target] (y-axis) with varying Target (x-axis) for releasing budget $k = 4000$.

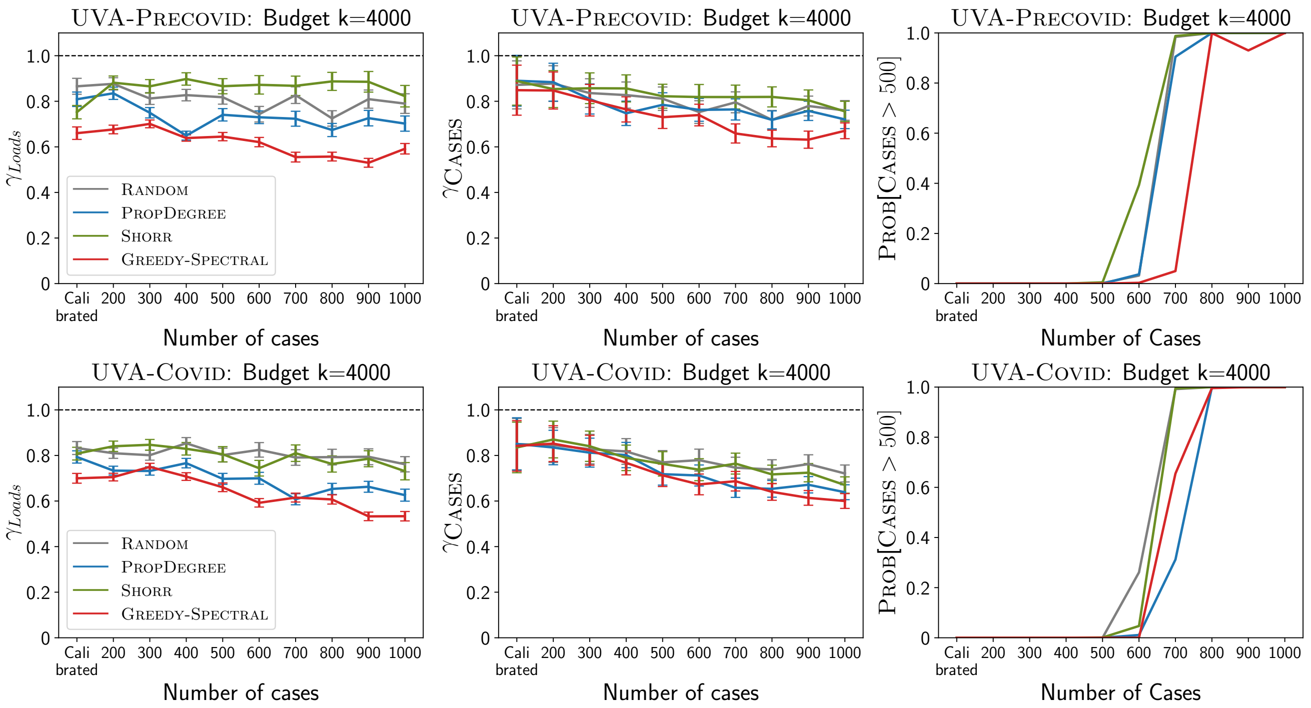

(a) γ_{Loads} (b) γCASES (c) PROB[CASES>500]

Supplementary Fig. 11. GREEDY-SPECTRAL achieves lower non-patient pathogen loads and number of new MRSA cases than other baselines with varying α under relaxed precaution and cleaning scenario for clearance problem. Top is for UVA-PRECOVID dataset, down is for UVA-COVID dataset. Here we fix the $k = 4000$. (a) Ratio of non-patient pathogen loads γ_{LOADS} (y-axis) with varying α (x-axis). (b) Ratio of new MRSA cases γ_{CASES} (y-axis) with varying α (x-axis). (c) PROB[CASES > 500] (y-axis) with varying α (x-axis) for budget $k = 4000$.

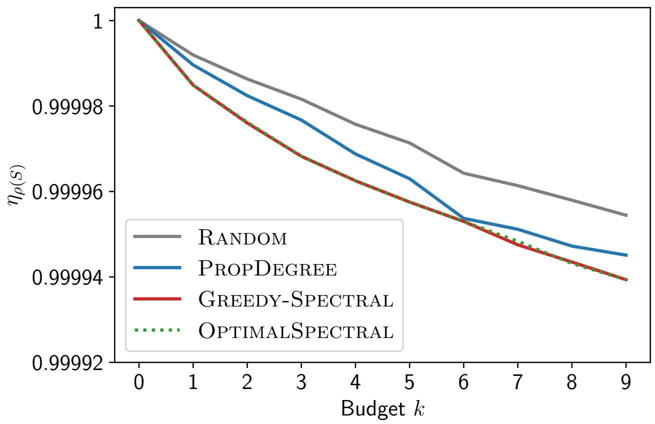

Supplementary Fig. 12. Results for minimizing $\rho(\boldsymbol{S})$ by minimizing $\rho(\boldsymbol{A})$ using Greedy-Spectral and OptimalSpectral. Here we show the change in $\eta_{\rho(\boldsymbol{S})}$ (y-axis) with increasing budget for isolation k (x-axis) by minimizing $\rho(\boldsymbol{A})$.

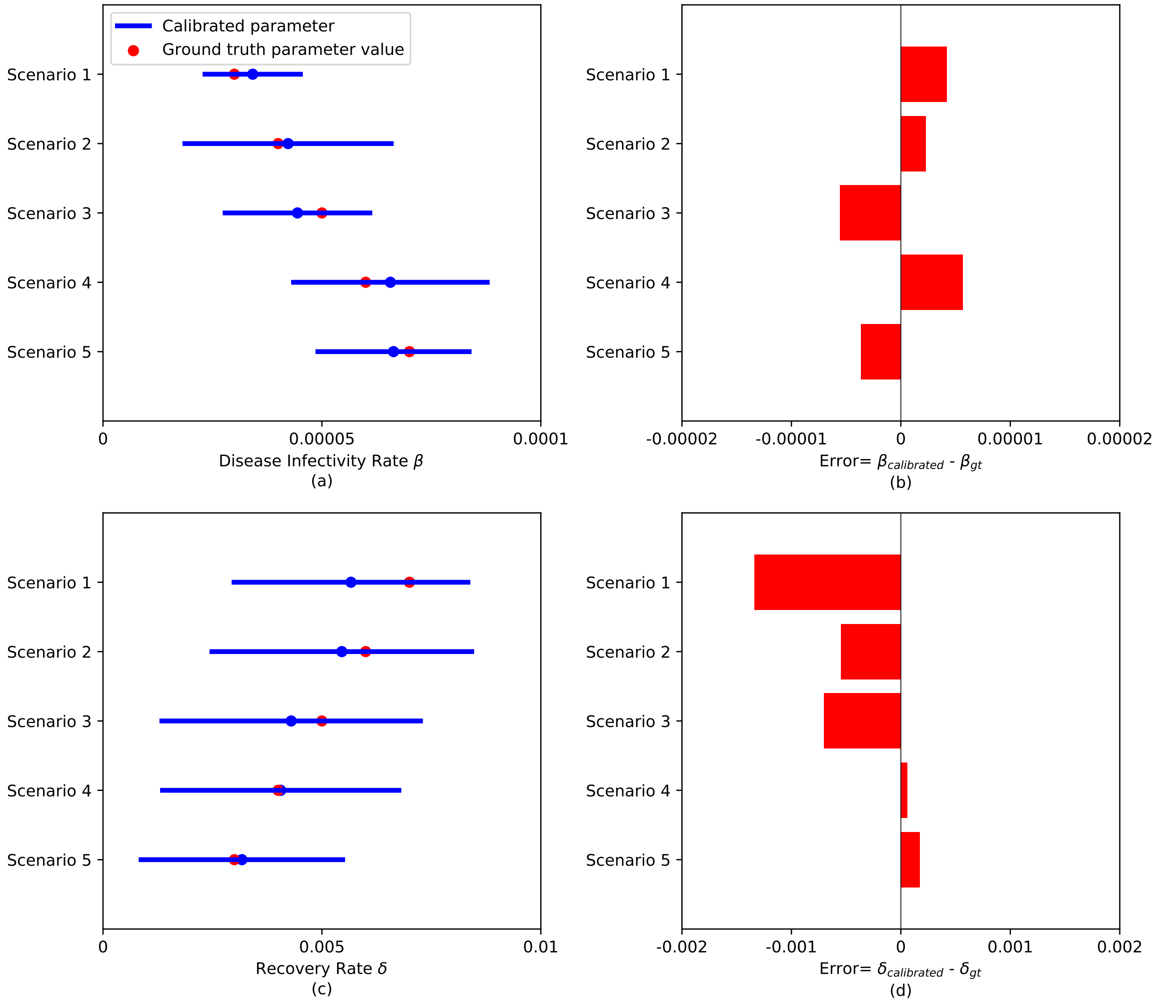

Supplementary Fig. 13. The calibration procedure effectively "recovers" the synthetic parameters by calibrating on the simulated curve using synthetic parameters. Left: the red dots are the ground-truth values of the synthetic parameters, and the blue dots (as well as error bars) are the average value and standard error of the calibrated parameters.

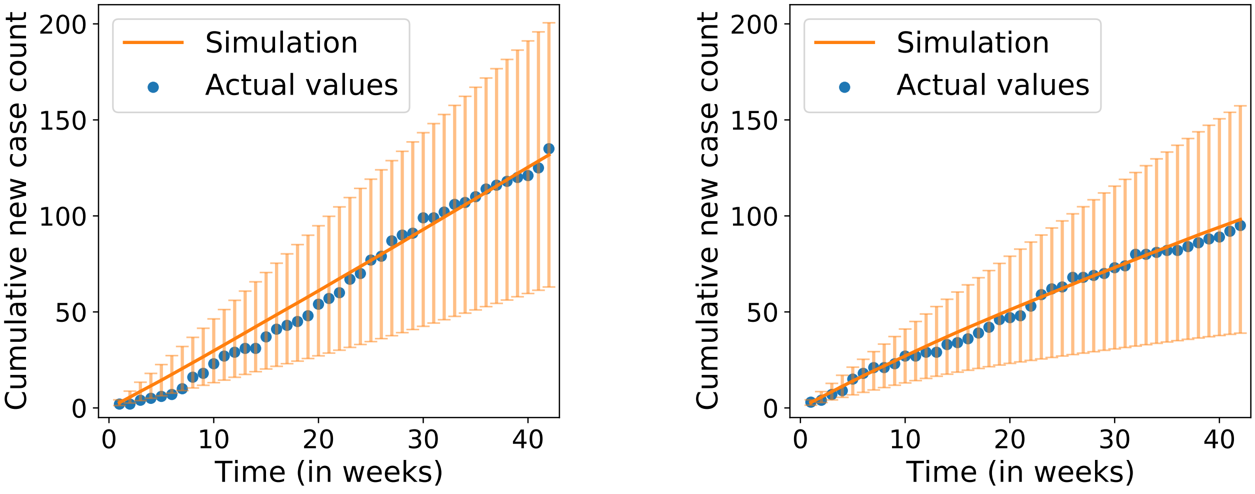

(a) UVA-PRECOVID

(b) UVA-POSTCOVID

Supplementary Fig. 14. Simulations on UVA testbeds averaged over 50 runs using calibrated parameters. The blue dots represent the actual values for the number of MRSA cases (cumulative values). The orange line and error bars represent the estimated mean value and standard error for the simulated number of MRSA cases using calibrated parameters. The average simulations closely match the real cumulative weekly new MRSA counts in both periods.

Supplementary Table 3. Basic statistics of real networks.

Notations	UVA-PRECOVID	UVA-COVID	LYON
T	294	294	4
P	8193	7600	29
H	4615	4526	46
L	595	596	0
N	13403	12722	75
M	2.16M	2.32M	1825

Supplementary Table 4. *Calibrated parameters for high precaution and cleaning scenario*

Parameter	UVA-PRECOVID	UVA-COVID
α	77.5553	61.9049
β	2.4585×10^{-5}	2.3088×10^{-5}
δ	0.0023	0.0020
ϕ_P	0.953	0.956
ϕ_H	0.600	0.779
ϕ_L	0.521	0.709
τ_{p2p}	0.0153	0.0133
τ_{p2h}	0.0112	0.0129
τ_{p2l}	0.0115	0.0154
τ_{h2p}	0.0153	0.0125
τ_{h2h}	0.0003	0.0014
τ_{h2l}	0.0041	0.0074
τ_{l2p}	0.0044	0.0060
τ_{l2h}	0.0097	0.0045

References

1. Stone, P. W. Economic burden of healthcare-associated infections: an american perspective. *Expert. review pharmacoeconomics & outcomes research* **9**, 417–422 (2009).
2. Sharma, A. *et al.* The cost of contact precautions: A systematic analysis. *Can. J. Infect. Control.* **35** (2020).
3. Roth, J. A. *et al.* Direct costs of a contact isolation day: a prospective cost analysis at a swiss university hospital. *infection control & hospital epidemiology* **39**, 101–103 (2018).
4. Valiquette, L., Chakra, C. N. A. & Laupland, K. B. Financial impact of health care-associated infections: When money talks. *Can. J. Infect. Dis. Med. Microbiol.* **25**, 71–74 (2014).
5. Howden, B. P. *et al.* Staphylococcus aureus host interactions and adaptation. *Nat. Rev. Microbiol.* 1–16 (2023).
6. Lowy, F. D. Staphylococcus aureus infections. *New Engl. journal medicine* **339**, 520–532 (1998).
7. Pei, S., Liljeros, F. & Shaman, J. Identifying asymptomatic spreaders of antimicrobial-resistant pathogens in hospital settings. *Proc. Natl. Acad. Sci.* **118**, e2111190118 (2021).
8. Sjoberg, A. M. *et al.* One health interprofessional stewardship to combat antimicrobial resistance. *Nat. Medicine* **29**, 512–513 (2023).
9. Marshall, C., Richards, M. & McBryde, E. Do active surveillance and contact precautions reduce mrsa acquisition? a prospective interrupted time series. *PloS one* **8**, e58112 (2013).
10. Hossain, A. D. *et al.* Effectiveness of contact tracing in the control of infectious diseases: a systematic review. *The Lancet Public Heal.* (2022).
11. Khdour, M. R. *et al.* Impact of antimicrobial stewardship programme on hospitalized patients at the intensive care unit: a prospective audit and feedback study. *Br. journal clinical pharmacology* **84**, 708–715 (2018).
12. George, P. & Morris, A. M. Pro/con debate: Should antimicrobial stewardship programs be adopted universally in the intensive care unit? *Critical Care* **14**, 1–6 (2010).
13. Haque, M. *et al.* Strategies to prevent healthcare-associated infections: a narrative overview. *Risk management healthcare policy* 1765–1780 (2020).
14. Bian, R., Koh, Y. S., Dobbie, G. & Divoli, A. Identifying top-k nodes in social networks: a survey. *ACM Comput. Surv. (CSUR)* **52**, 1–33 (2019).
15. Chen, W., Wang, Y. & Yang, S. Efficient influence maximization in social networks. In *Proceedings of the 15th ACM SIGKDD international conference on Knowledge discovery and data mining*, 199–208 (2009).
16. Chen, S. & He, K. Influence maximization on signed social networks with integrated pagerank. In *2015 IEEE International Conference on Smart City/SocialCom/SustainCom (SmartCity)*, 289–292 (IEEE, 2015).
17. Subbian, K., Aggarwal, C. & Srivastava, J. Content-centric flow mining for influence analysis in social streams. In *Proceedings of the 22nd ACM international conference on Information & Knowledge Management*, 841–846 (2013).
18. Kong, L. *et al.* In *International Conference on Learning Representations* (2023).
19. Zhu, J.-M., Wang, L., Liu, J.-B. *et al.* Eradication of ebola based on dynamic programming. *Comput. Math. Methods Medicine* **2016** (2016).
20. England, P. H. Guidance for stepdown of infection control precautions and discharging covid-190 patients (2020).
21. Siegel, J. D., Rhinehart, E., Jackson, M. & Chiarello, L. 2007 guideline for isolation precautions: preventing transmission of infectious agents in health care settings. *Am. journal infection control* **35**, S65–S164 (2007).
22. Chakrabarti, D., Wang, Y., Wang, C., Leskovec, J. & Faloutsos, C. Epidemic thresholds in real networks. *ACM Transactions on Inf. Syst. Secur. (TISSEC)* **10**, 1–26 (2008).
23. Prakash, B. A., Tong, H., Valler, N., Faloutsos, M. & Faloutsos, C. Virus propagation on time-varying networks: Theory and immunization algorithms. In *Joint European Conference on Machine Learning and Knowledge Discovery in Databases*, 99–114 (Springer, 2010).
24. Prakash, B. A., Chakrabarti, D., Faloutsos, M., Valler, N. & Faloutsos, C. Threshold conditions for arbitrary cascade models on arbitrary networks. In *2011 IEEE 11th International Conference on Data Mining*, 537–546 (IEEE Computer Society, 2011).
25. Jang, H. *et al.* Evaluating architectural changes to alter pathogen dynamics in a dialysis unit. In *2019 IEEE/ACM International Conference on Advances in Social Networks Analysis and Mining (ASONAM)*, 961–968 (IEEE, 2019).

26. Plipat, N., Spicknall, I. H., Koopman, J. S. & Eisenberg, J. N. The dynamics of methicillin-resistant staphylococcus aureus exposure in a hospital model and the potential for environmental intervention. *BMC infectious diseases* **13**, 1–11 (2013).

27. Chandrasekaran, S. & Jiang, S. C. A dose response model for staphylococcus aureus. *Sci. Reports* **11**, 1–10 (2021).

28. Brouwer, A. F., Weir, M. H., Eisenberg, M. C., Meza, R. & Eisenberg, J. N. Dose-response relationships for environmentally mediated infectious disease transmission models. *PLoS computational biology* **13**, e1005481 (2017).

29. Rubin, M. A. *et al.* A simulation-based assessment of strategies to control clostridium difficile transmission and infection. *PloS one* **8**, e80671 (2013).

30. Nelson, R. E. *et al.* An economic analysis of strategies to control clostridium difficile transmission and infection using an agent-based simulation model. *PloS one* **11**, e0152248 (2016).

31. Adhikari, B., Lewis, B., Vullikanti, A., Jiménez, J. M. & Prakash, B. A. Fast and near-optimal monitoring for healthcare acquired infection outbreaks. *PLoS computational biology* **15**, e1007284 (2019).

32. Coia, J. *et al.* Guidelines for the control and prevention of meticillin-resistant staphylococcus aureus (mrsa) in healthcare facilities. *J. hospital infection* **63**, S1–S44 (2006).

33. of Disease Prevention, O. & Promotion, H. Health care-associated infections. https://www.cdc.gov/mrsa/community/environment/index.htm.

34. Hethcote, H. W. The mathematics of infectious diseases. *SIAM review* **42**, 599–653 (2000).

35. Sayama, H. *Introduction to the modeling and analysis of complex systems* (Open SUNY Textbooks, 2015).

36. Pujol, J. M., Eisenberg, J. E., Haas, C. N. & Koopman, J. S. The effect of ongoing exposure dynamics in dose response relationships. *PLoS computational biology* **5**, e1000399 (2009).

37. Schmidt, K. A. & Ostfeld, R. S. Biodiversity and the dilution effect in disease ecology. *Ecology* **82**, 609–619 (2001).

38. Kempe, D., Kleinberg, J. & Tardos, É. Maximizing the spread of influence through a social network. In *Proceedings of the ninth ACM SIGKDD international conference on Knowledge discovery and data mining*, 137–146 (2003).

39. Bartsch, S. M. *et al.* Modeling interventions to reduce the spread of multidrug-resistant organisms between health care facilities in a region. *JAMA Netw. Open* **4**, e2119212–e2119212 (2021).

40. Garey, M. R. & Johnson, D. S. *Computers and intractability*, vol. 174 (freeman San Francisco, 1979).

41. Van Mieghem, P. *et al.* Decreasing the spectral radius of a graph by link removals. *Phys. Rev. E* **84**, 016101 (2011).

42. Horn, R. A. & Johnson, C. R. *Matrix analysis* (Cambridge university press, 2012).

43. Alman, J. & Williams, V. V. A refined laser method and faster matrix multiplication (2020). 2010.05846.

44. Vanhems, P. *et al.* Estimating potential infection transmission routes in hospital wards using wearable proximity sensors. *PloS one* **8**, e73970 (2013).

45. Valdano, E., Poletto, C., Boëlle, P.-Y. & Colizza, V. Reorganization of nurse scheduling reduces the risk of healthcare associated infections. *Sci. reports* **11**, 1–12 (2021).

46. Shorr, A. F. *et al.* A risk score for identifying methicillin-resistant staphylococcus aureus in patients presenting to the hospital with pneumonia. *BMC infectious diseases* **13**, 1–7 (2013).

47. Dutta, R., Schoengens, M., Onnela, J.-P. & Mira, A. Abcpy: A user-friendly, extensible, and parallel library for approximate bayesian computation. In *Proceedings of the platform for advanced scientific computing conference*, 1–9 (2017).
